# C3NA: correlation and consensus-based cross-taxonomy network analysis for compositional microbial data

**DOI:** 10.1186/s12859-022-05027-9

**Published:** 2022-11-08

**Authors:** Kuncheng Song, Yi-Hui Zhou

**Affiliations:** Bioinformatics Research Center, Biological Sciences, North Carolina State University, Raleigh, NC USA

**Keywords:** Co-occurrence network analysis, Microbiome, R package, Consensus clustering, Module preservation analysis

## Abstract

**Background:**

Studying the co-occurrence network structure of microbial samples is one of the critical approaches to understanding the perplexing and delicate relationship between the microbe, host, and diseases. It is also critical to develop a tool for investigating co-occurrence networks and differential abundance analyses to reveal the disease-related taxa–taxa relationship. In addition, it is also necessary to tighten the co-occurrence network into smaller modules to increase the ability for functional annotation and interpretability of  these taxa-taxa relationships.  Also, it is critical to retain the phylogenetic relationship among the taxa to identify differential abundance patterns, which can be used to resolve contradicting functions reported by different studies.

**Results:**

In this article, we present Correlation and Consensus-based Cross-taxonomy Network Analysis (C3NA), a user-friendly R package for investigating compositional microbial sequencing data to identify and compare co-occurrence patterns across different taxonomic levels. C3NA contains two interactive graphic user interfaces (Shiny applications), one of them dedicated to the comparison between two diagnoses, e.g., disease versus control. We used C3NA to analyze two well-studied diseases, colorectal cancer, and Crohn’s disease. We discovered clusters of study and disease-dependent taxa that overlap with known functional taxa studied by other discovery studies and differential abundance analyses.

**Conclusion:**

C3NA offers a new microbial data analyses pipeline for refined and enriched taxa–taxa co-occurrence network analyses, and the usability was further expanded via the built-in Shiny applications for interactive investigation.

**Supplementary Information:**

The online version contains supplementary material available at 10.1186/s12859-022-05027-9.

## Background

In recent years, researchers have discovered specific yet complex links between the human gut microbial ecology and diseases such as colorectal cancer [1–3] and inflammatory disorders [4–6]. According to a widely recognized model for the microbe-human interaction, dysbiosis of gut microbiota is associated with the development of illnesses [7]. Different microorganisms will thrive or decline depending on the host illness progression, medicine, food, and other variables. Studies on the essential bacteria linked with a disease have shown various integral microbes that play vital roles in illness progression. These findings aided in the development of predictive models and targeted therapies. 16S ribosomal RNA amplicon sequencing is the most cost-effective and accessible way to obtain microbial compositional data. It is supported by established downstream taxonomic classification pipelines/software, curated 16S ribosomal reference databases, and statistical methods designed to analyze microbial compositional data [8].

Currently, two of the frequently used methods for detecting important microbes are differential abundance (DA) analysis and co-occurrence network analysis. DA method is a popular choice for finding specific microbial taxa associated with ill or healthy people, and co-occurrence networks aim to decipher the taxa–taxa co-occurrence patterns for unique disease- enriched or depleted pattern discovery. The concordances of identified differentially abundant taxa are one of the main challenges in DA approaches. While numerous methods can be used to evaluate consensus taxa, the results are influenced by sample/study variances and filtering criteria [9]. Thus, one of the optimal solutions for maximizing DA methods is to utilize different DA results as a guide to investigate the structural difference between the disease and control co-occurrence network structure. Previous co-occurrence networks provided helpful information about taxonomic and phenotypic associations [10–12], and found significant taxa–taxa correlations, and the resultant networks are usually condensed and highly connected. In addition, the resulted nodes/taxa are usually concentrated on a specific taxonomic level (E.g., species, genus, or operational taxonomic units (OTUs)).

Depending on the sample size and experimental design, the resolution on more refined taxonomic levels, such as genus and species assignment, might be negatively affected due to missing or unknown classification. Thus, studying a single taxonomic level might only offer a single aspect of the microbial community, and it is of utmost importance to study the structure from cross-taxonomic count matrices that encompass many taxonomic levels to ensure we capture the relationship among and between the parent and their children taxa. Our results from C3NA have shown that it is not necessary for the more refined taxonomic assignment of a particular taxon to share common higher taxonomic assignments. This differential assignment based on the abundance pattern motivated us to persuade a cross-taxonomy approach to categorize the taxa into different modules from the taxa–taxa correlation patterns.

To account for the cross-taxonomy design and the unique microbial compositional structure, we utilized the Sparse Correlation for Compositional data (SparCC) correlation to obtain the cross-taxonomy correlations [13]. We also employed a consensus-based approach to combine the modular information from a range of patterns to strengthen our confidence in the optimal number of taxa clusters, which suggests shared functional inferences. In addition, C3NA calculates the modular information from each of the conditions (e.g., colorectal cancer and control) independently, and the retained modular information will then be compared interactively on our built-in Shiny application. This approach intends to minimize the study-dependent correlations, as the high taxa–taxa correlation that is shared between the disease and control samples might not be the important taxa that enriched or depleted caused by the disease.

Lastly, C3NA is able to encounter many of the challenges we have mentioned, and it is developed as a user-friendly and open-source R-package that includes data processing and interactive visualization functionalities. The goal of the C3NA is to maximize the available biologically inferable information in terms of taxonomic assignments across Phylum, Class, Order, Family, Genus, and Species levels via co-occurrence network analysis to extract optimal numbers of co-occurring taxa modules with similar taxonomic abundance patterns. With the ability to concurrently view the DA and modularized co-occurrence network, C3NA demonstrates great potential for identifying and assigning taxa and groups of taxa that are positively or negatively related to a particular disease.

## Methods

### Raw 16S rRNA data source and analysis pipeline information

We evaluated C3NA using two colorectal cancer (CRC) datasets and two inflammatory bowel diseases with Crohn’s Disease (CD) 16S rRNA datasets. The first CRC dataset labeled as “cancer” was from PRJNA290926 [14], and the second CRC dataset labeled as “cancer2” was extracted from PRJEB6070 [15]. For both CRC datasets, we only used the samples labeled as “Cancer” and “Normal.“ The first Crohn’s disease dataset was downloaded from PRJEB13679 [16], and we used the “CD” and “no” as the case and control, respectively. The second Crohn’s disease dataset was from the IBDMDB [17] website, and we used the “CD” and “non-IBD” as the case and control, respectively. The data were loaded to QIIME2 according to their respective study design following the DADA2 algorithm pipeline [18]. All assignments used the same SILVA 138 [19] reference under the QIIME2 (version 2021.4) environment [8]. The dataset information is shown in Additional file 1: Table S1.

### Microbial data processing

**Fig. 1:**
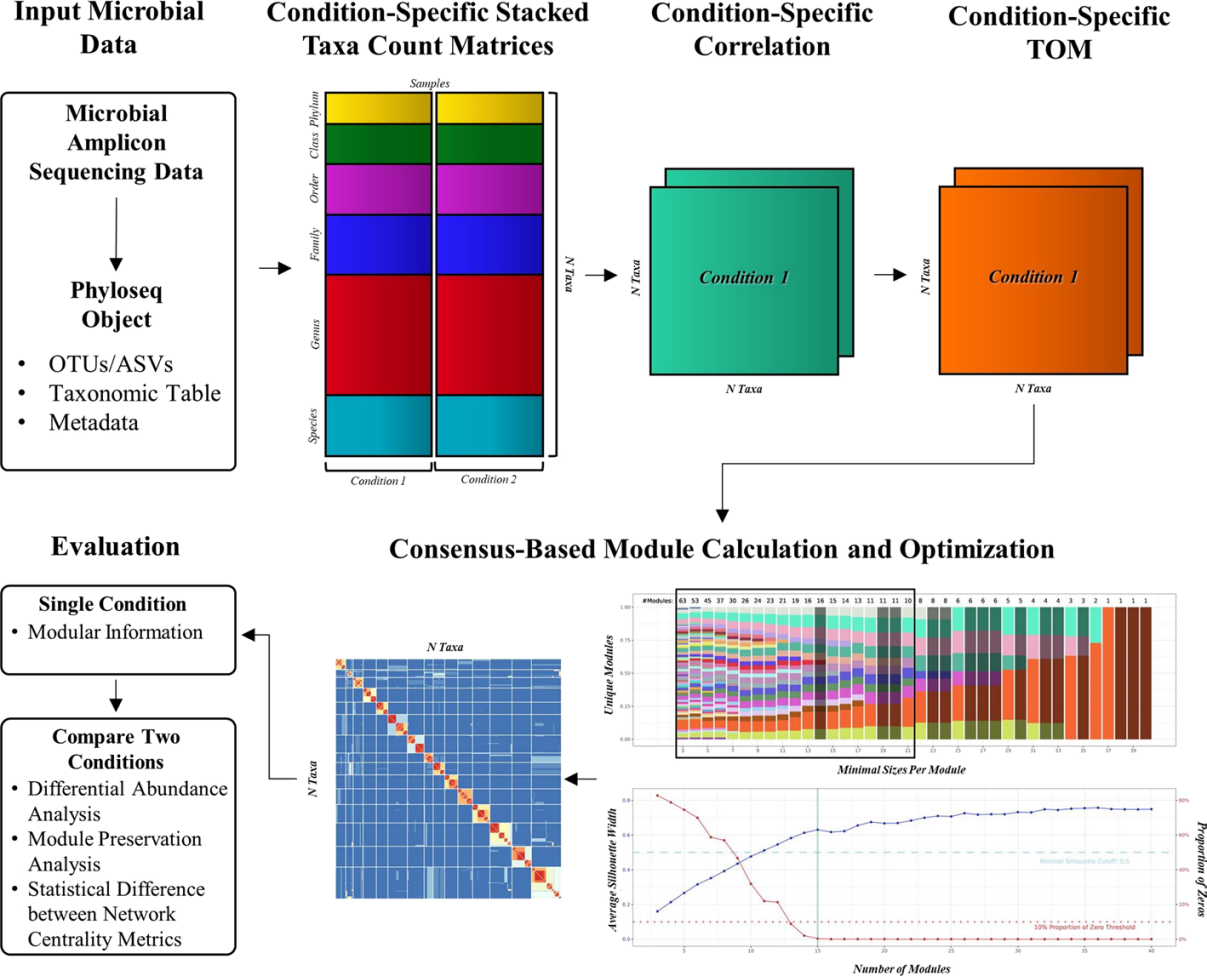
C3NA framework illustrated for two conditions comparison examples. For every phenotype/diagnosis, a condition-specific Phyloseq will be used as input to generate the condition-specific stacked-taxa count matrix by combining Phylum, Class, Order, Family, Genus, and Species-level raw count matrix. Then, the matrix undergoes SparCC correlation calculation with 1000 bootstraps followed by the topological overlap matrix (TOM) calculation under the “signed” network setting. Next, the dissimilarity TOM matrix (1-TOM) is used for hierarchical clustering with a range of minimal taxa per module (3–40) to extract a range of clustering patterns. A selected range of patterns is used to generate a consensus matrix, in which the intra-modular connections are the key taxa–taxa correlations we focus on in the subsequent network analysis. When comparing two conditions, module preservation analyses and other statistical methods are performed

Many established pipelines are available for processing the 16S rRNA amplicon sequencing to summarize the raw sequencing data into taxonomic profiles, such as the operational taxonomic units (OTUs) and amplicon sequencing variants (ASVs). For C3NA, we utilized the QIIME2 pipeline with the DADA2 algorithm to generate the ASVs from each of the four datasets [18, 20]. Regardless of the methods and reference database, the resulting taxonomic profile includes an ASVs table, a taxonomic table, and a metadata table. To ensure the accuracy of these data prior to loading into the C3NA pipeline, we utilize the Phyloseq R package to ensure the correct formatting of these tables, and the Phyloseq object is the initial input for the C3NA pipeline [21]. Lastly, the user needs to run different conditions separately by sub-setting the Phyloseq object and running the pipeline described in Fig. 1 individually. The C3NA pipeline is split into the following five sections.

#### Generating condition-specific stacked-taxa count matrices

Prior to generating the condition-specific stacked-taxa count matrices, the user should utilize the Phyloseq functions to filter out a single condition. Also, we recommended removing samples with a library size of fewer than 1000 reads as these samples are known to suffer from low-quality issues in terms of microbial diversity as well as sequencing-related issues [22]. Generally, there should be six levels of taxonomic levels, Phylum, Class, Order, Family, Genus, and Species for each of the ASVs, and for each of them, we will sum the ASV count matrices to their respective assignments and then stack them to form a stacked-taxa table. Lastly, as we are focusing on higher taxonomic levels than OTUs/ASVs by summing the OTUs/ASVs counts, we filtered out the taxa that did not present in at least 10% of the samples to remove rare taxonomic assignments [13]. This filtering criteria coincide with one of the assumptions for SparCC in which the kept taxa are assumed to be present among samples, and this approach also drastically reduces the computational complexity with the reduced number of taxa. For each of the conditions within the study, a condition-specific matrix is created by applying the aforementioned filtering procedures.

#### Condition-specific SparCC correlation

We employed Sparse Correlation for Compositional data (SparCC) correlation with 1000 bootstrap settings employed using the SPIEC-EASI R Package [13, 23]. The bootstrap resulted in a correlation value between each taxa pair and a *p*-value, and the Benjamini-Hochberg (BH) method will subsequently be applied to adjust for multiple testing. We also investigated the effects of different numbers of bootstraps in Additional file 1: Fig. S10, and undertaking at least ten bootstraps is recommended.

We further validated the stability of the SparCC on how the stacked-taxa affects the taxa–taxa correlations (detailed in Additional file 1: Results and Fig. S1). We investigated the impact of using the stacked-taxa correlation compared to the single-taxonomic level correlations and found minimal differences for the taxa–taxa pairs, especially for the correlations above 0.2. There is no drastic difference between the stacked-taxa and single-taxonomic level correlations. As a result, we recommended a minimal correlation cutoff at 0.2 to remove uncertain and weak correlations, and the users can further filter the correlation strength in the shiny application.

We also compared SparCC results with COAT [24] and Pearson [25]. The Pearson results are drastically different from that of the COAT and SparCC as Pearson correlations could not natively handle compositional data (detailed in Additional file 1: Results and Fig. S12). The comparison between COAT and SparCC reveals a high correlation (r of 0.904), with COAT generally having slightly higher correlation values than SparCC for the same taxa–taxa pairs (Additional file 1: Fig. S13A). Moreover, we compared the incorporation of DESeq2 methods to balance the Case and Control samples prior to running the correlation methods, and different correlation methods can generate different results, with most of the highly correlated taxa–taxa pairs shared among all methods (detailed in Additional file 1: Results, Supplementary Tables S2, Figs. S13A, B and S14A, B). The users are advised to investigate the resulting correlation distribution before setting the minimal correlation cutoffs.

#### Condition-specific topological overlap matrix (TOM)

The Topological Overlap Matrix (TOM ) is constructed from the correlation matrix under the signed network setting using the WGCNA R package [26]. The signed network is chosen because negative correlations should not be interpreted the same as positive correlations, as they carry different biological meanings. The result in the TOM matrix represents the network connections’ strength, especially for spurious connections [27]. As a result of this procedure, our analyses will focus on positive taxa–taxa correlations, which will be used to generate modules, and all correlation values are stored and available for extraction if needed for other analyses.

#### Consensus-based module determination and optimization

**Fig. 2:**
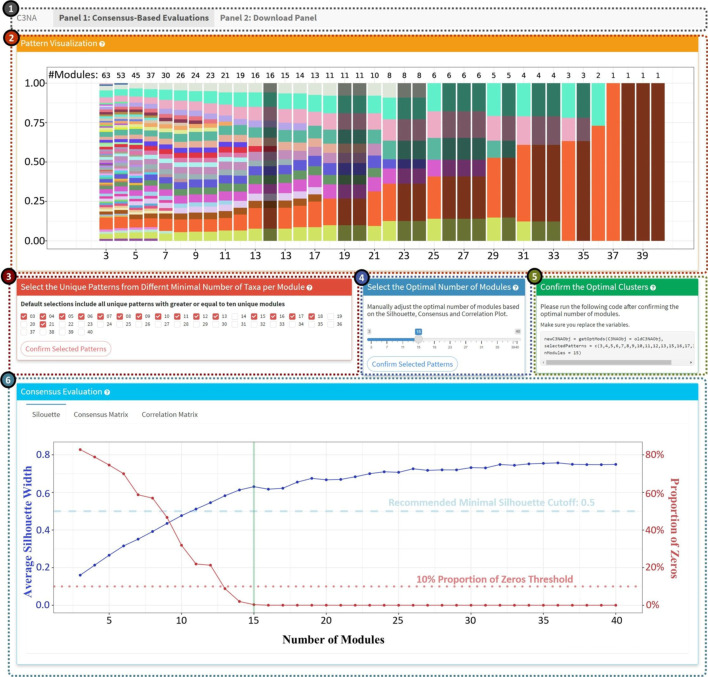
Consensus-based approach guide for module identification shiny application. **1**. Shiny panel navigation, including the ‘Consensus-Based Evaluations’ and ‘Download Panel’. **2**. Unique patterns of the modules based on a range of a minimal number of taxa per module. The darkened columns highlighted the duplicated patterns which are not included in the consensus-based evaluations. **3.** Manually select the pattern columns. The default will select all unique patterns with modules greater or equal to ten. **4**. Manually select the optimal number of modules based on the silhouette, consensus, and correlation plots below. **(5)** Generate the code the user should run after confirming the optimally selected patterns and the optimal number of modules. **(6)** Plot display panel including silhouette, consensus, and correlation plots. *Note: *the font size in the image has been adjusted for this publication

We obtained the dissimilarity TOM via (1-TOM) and used the complete linkage hierarchical clustering to classify the taxa into different modules. We examined a range of a minimal number of taxa per module (3 and 40), displayed as different color patterns in box 2 of Fig. 2. There is an apparent and dynamic change with decreasing number of unique modules. The module became more stable as the number of unique modules reduced to less than 10, where more repetitive module patterns emerged. By default, C3NA combines all the unique module patterns equal to or greater than ten modules (Fig. 2, Additional file 1: Results and Fig. S8). Our investigation from C3NA has shown that it is important to focus on the region in which the resulted in different modular patterns (with decreasing number of unique modules) with each incrementing minimal module size. We investigated the difference between choosing different numbers of unique patterns and the optimal number of clusters and discovered that positive correlations within modules are stable when the user selects modules that still undergo shifting in terms of the number of resulting modules. A more detailed investigation is in the Additional file 1: Results.

Once the module patterns are generated, the consensus-based module determination uses the Cluster-based Similarity Partitioning Algorithm (CSPA) [28]. We extract each taxa–taxa modular assignment and create an individual binary similarity matrix with the presence of taxa–taxa pairs in the same module as 1, otherwise 0. The consensus matrix is obtained via averaging all these similarity matrices.

The next step is to obtain the optimal number of clustering based on the consensus matrix. Two parameters are used to determine the optimal number of clustering. Firstly, we utilize the proportion of zeros per module; this proportion should be below 10%. Secondly, we calculated the average silhouette width for each clustering based on the consensus matrix, and we will choose the first local maximum, representing the first drop in the average silhouette score. We use the corresponding modular membership to construct the intra-modular taxa modules. The module patterns, silhouette results, consensus, and correlation matrices for our examined datasets with different taxonomic assignment methods are illustrated in Additional file 1: Fig. S9. Once the module is determined, the user can utilize our shiny application to investigate the single condition taxa–taxa relationship or compare two conditions to determine the preserved or perturbed modules.

The user will be guided through this investigation on our built-in shiny application, as shown in Fig. 2.

#### Single condition extraction and two conditions comparison

The results from the previous step will be stored in a single R object representing the modular information from a single condition. We will combine both conditions’ results to evaluate disease and control samples and use module preservation analysis to assess the differential taxa in network structure alteration between the two conditions. We use the *Z*_*Summary*_, a composite preservation statistic proposed by Langfelder et al., to evaluate the module preservation between the disease and control. *Z*_*Summary*_ compared the connectivities of the intramodular nodes and the highly connected nodes between the comparison groups. The *medianRank*, which is less sensitive to module size, is also selected to assist the definition of preserved modules [29–31]. Ideally, the higher the *Z*_*Summary*_ and the lower the *medianRank*, the more preserved the module is.

Moreover, as microbial modules can be tiny, users should distinguish a more extensive and more diverse module from a smaller taxon with very similar phylogenetic information, i.e., taxa from the same phylogenetic branch. From a biological point of view, high and low preservation modules are critical. The high preservation module contains the connections between two conditions, and the standard preservation modules have modules and elements that are either of no interest or condition-specific perturbation. However, it is essential to evaluate all modules, and modules with differentially abundant taxa are often significant.

**Fig. 3:**
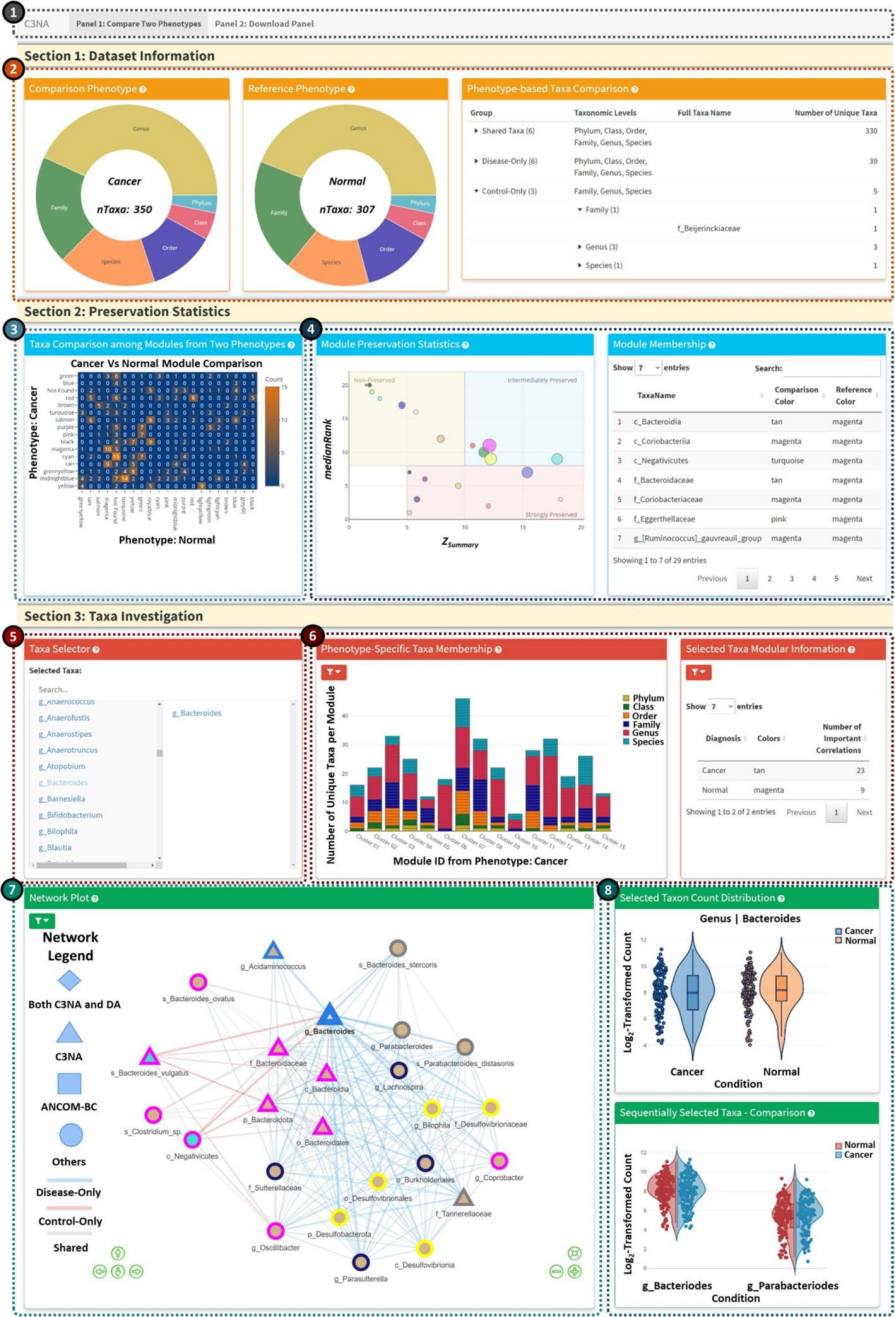
Shiny application for two conditions comparison. **1**. Shiny panel navigation, including the ‘Compare Two Conditions’ and ‘Download Panel’. **2**. Condition-based information from the two conditions, including shared and different taxa from various taxonomic levels. **3**. Module-based taxa comparison between the two conditions. **4**. Module preservation investigation, the left plot shows the Median Rank Vs. Z Summary with the node size reflects the number of taxa in the respective module. The right plot is an interactive table from reacting to the click of the module preservation plot. **5**. Taxa selector for the interactive display in Panels 6–8. The user can select multiple taxa from the left columns (including all significant taxa from both conditions). To remove a selected taxon, the user can click the taxon from the right column. **6**. Bar plot for the taxa membership among the modules from either condition. The user can switch between viewing all taxa or the selected taxa from the ‘Taxa Selector’. For better display, the bar plot will only display the select taxon and its children’s taxonomic level taxa. Please click on the left filter icon to view plot-based options. **7**. Network plot based on the selected taxa. Multiple options are available from the top left filter icon. **8**. Single and sequentially clicked taxa and their respective log2-transformed count from both conditions. *Note: *the font size in the image has been adjusted for this publication

Once the two conditions comparison results are calculated, the users can utilize another Shiny application, as shown in Fig. 3, to evaluate the results interactively.

### Evaluation of the optimal number of clusters

Two significant considerations determine the appropriate number of clusters. The first is the distinct patterns of modules picked from a different minimum number of taxa per module for building the consensus matrix. The silhouette width evaluation of the consensus matrix with hierarchical clustering is the second factor. We investigated how different selections of these two parameters affect the downstream analysis in terms of intra-modular correlations, and our results show that it is essential to select a pattern with less dynamic region of the module patterns, around ten modules (Additional file 1: Fig. S8). Moreover, for optimal cluster selection, it is vital to choose a minimum number as the curve turns into a plateau region. Once these two are selected carefully, the resulting intra-modular correlations are very similar and should not drastically affect the preservation and network analysis. A complete investigation is in Additional file 1: Result.

### Network centrality metrics

There are three network centrality metrics used by the C3NA, degree centrality, closeness centrality, and transitivity using the igraph R package (version 1.2.8) [32]. The purpose of these parameters is to infer the significance of the taxon between the comparing conditions. We choose the normalized version for the degree and closeness to account for the total number of vertices in the graph, making the results more comparable. We also calculated the local transitivity for the node’s importance within the local network. We extract the corresponding intra-modular members from the two conditions for each taxon to construct a network. We subsequently calculated the three parameters with and without the selected taxon, then used the paired-sample Wilcoxon test to compare the changes among these three network metrics. We define the influential taxon as one with at least one statistically significant difference after BH-adjusted *p*-values ≤ 0.05.

### Intra-modular evaluation

For each of the modules, we will keep the taxa–taxa correlation greater or equal to 0.2 with a BH-adjusted *p*-value no higher than 0.05. Next, we obtain the threshold by comparing correlations at the stack-taxonomic and single-taxonomic levels (Additional file 1: Results). The combination of these two parameters will help estimate the influential taxa within each module.

### Differential abundance analyses

In our analyses, we chose three validated DA methods and executed them on each of the six taxonomic levels, including ANOVA-Like Differential Gene Expression Analysis (ALDEx2) [33], Analysis of Compositions of Microbiomes with Bias Correction (ANCOM-BC) [34], and Multivariable Association Discovery (MaAsLin2) [35]. As expected, only a small number of taxa were consistently identified by the methods from different clustering methods, and the proportion of the consensus taxa among the OTUs/ASVs clustering methods is much larger (Additional file 1: Results). We utilized the recommended 10% prevalence filtering for each of the Phylum to Species-level assignments prior to running the methods on our dataset to obtain more robust results [36]. Each differential abundance analysis was performed between the disease and control samples with a binary outcome. The DA methods’ settings are detailed in the Additional file 1: Results.

## Results

### Differential abundance analyses

There is a clear pattern of inconsistency in which taxa are DA among the three DA methods evaluated, and they are summarized in Additional file 1: Figs. S2–S5 for the four datasets we examined. In addition, ANCOM-BC obtained the most DA taxa compared to the other methods.

### Filtered taxa comparison

**Fig. 4:**
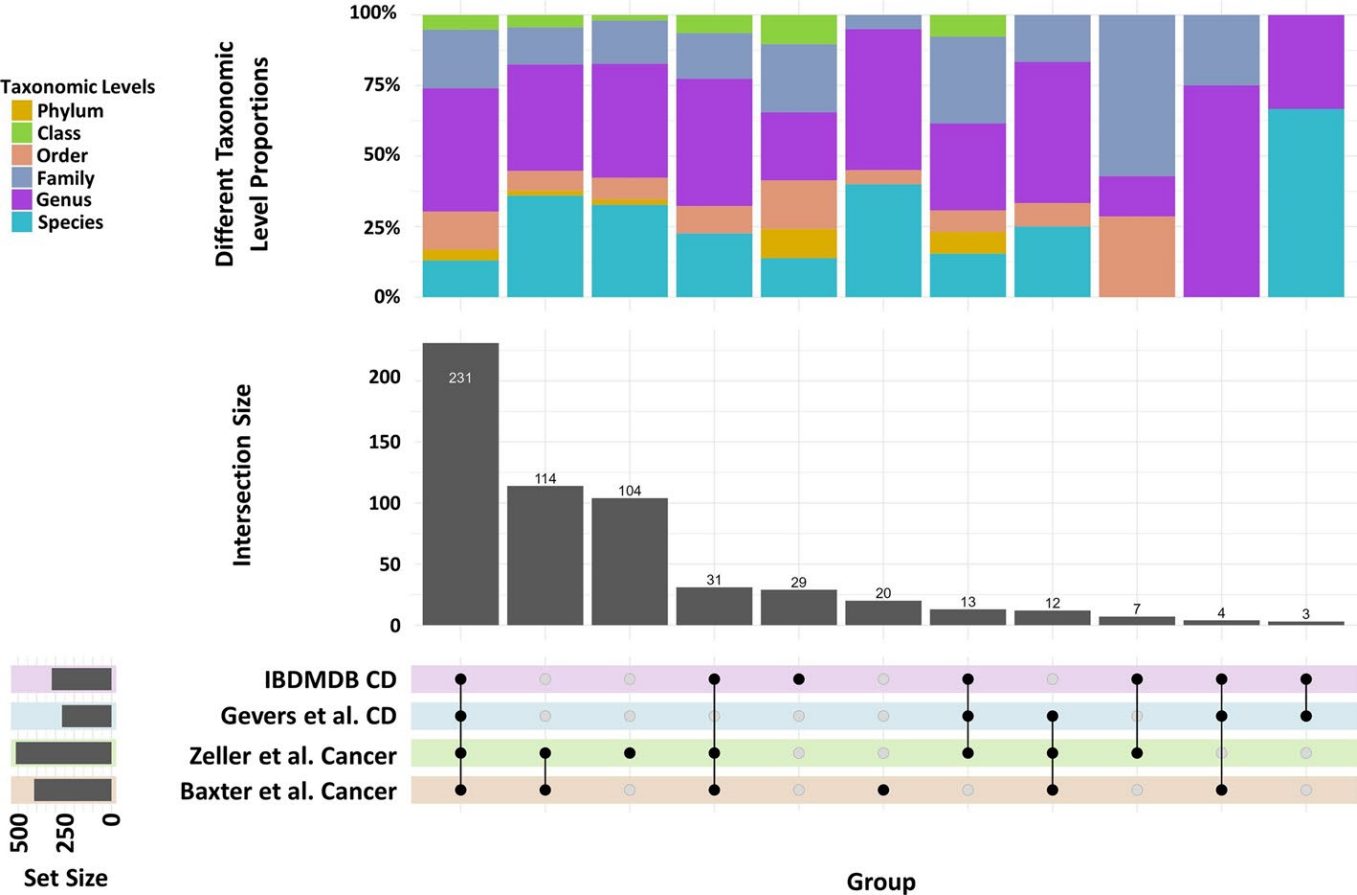
The shared taxa patterns among the studies and taxonomic assignment methods. The top bar plot illustrates the relative number of taxa from each of the six taxonomic levels. The bottom upset plot and the interaction plot illustrate the number of shared taxa and patterns among the examined datasets and taxonomic assignment methods

We apply 10% sample prevalence filtering is used to remove rare taxa from each condition. We investigated the filtered taxa across all studies shown in Additional file 1: Figs. S6 and S7. The remaining taxa showed consistency and specificity toward the disease. There are more taxa left with CRC than CD, which conforms with the lower bacterial diversity in CRC compared to CD (Fig. 4) [35].

### Identification of condition-specific taxa–taxa correlations

**Fig. 5:**
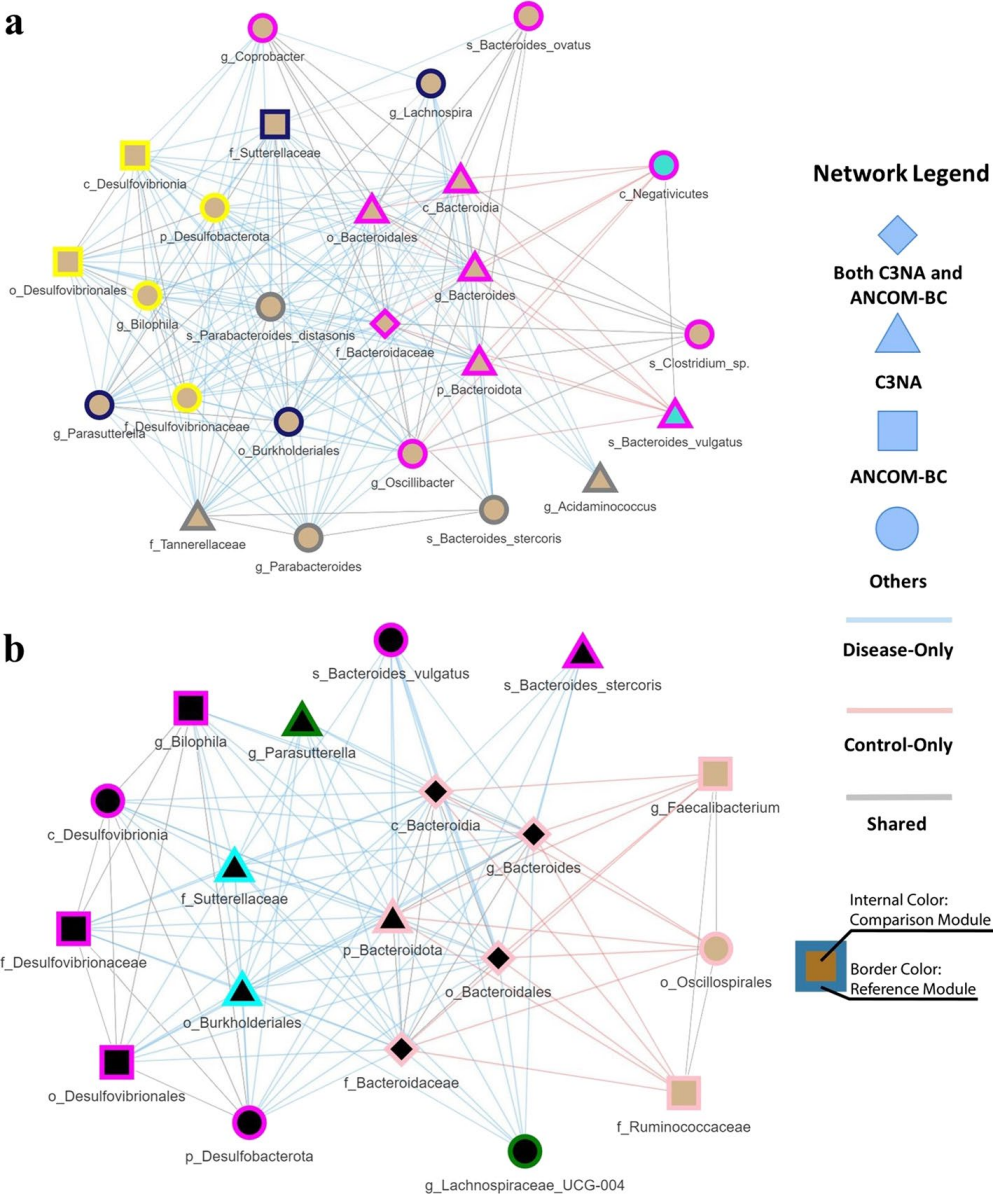
The networks were created from selective Taxa. **a** Taxa related to Genus *Bacteroides* from Baxter et al. with the condition comparison between colorectal cancer and control. **b** Taxa that related to Genus *Bacteroides* from Gevers et al. with the comparison of the conditions between Crohn’s disease and control

One of the critical findings from C3NA among these four datasets is the connection between the Genus *Bacteroides* and *Parabacteroides*, which has an intra-modular correlation found among CRC and is absent among CD datasets (Fig. 5a, b). This correlation between Bacteroides and Parabacteroides is 0.641 and 0.416 for CRC and Healthy samples, respectively. The consensus-based clustering groups them into the same module in CRC with a consensus of 1 and 0 for the control and an indication that their abundance patterns are altered between colorectal cancer and control samples. The interactive network plot illustrated in Fig. 5a highlights condition-specific taxa–taxa correlations by using blue, red, and gray for disease-only, control-only, and shared taxa–taxa correlations. *Bacteroides* are known to be enriched among colorectal cancer cases among other Genus-level assignments, including *Bilophila *[37], *Coprobacter*[37], and *Acidaminococcus *[38]. For the *Bacteriodes*-related Genus assignments in Crohn’s disease, C3NA identified three related Genus-level assignments, including *Bilophila *[39], *Parasutterella *[40], and *Lachnospiraceae *[41]. In each of the diseases, C3NA clustered these Genus-level assignments into the same module (same inner node colors) with disease-only correlation connecting between them, indicating similar abundance patterns among them in the respective diseases. These modules with the corresponding disease-only correlations can assist researchers in validating and identifying new biomarkers based on known taxa related to a given disease.

**Fig. 6:**
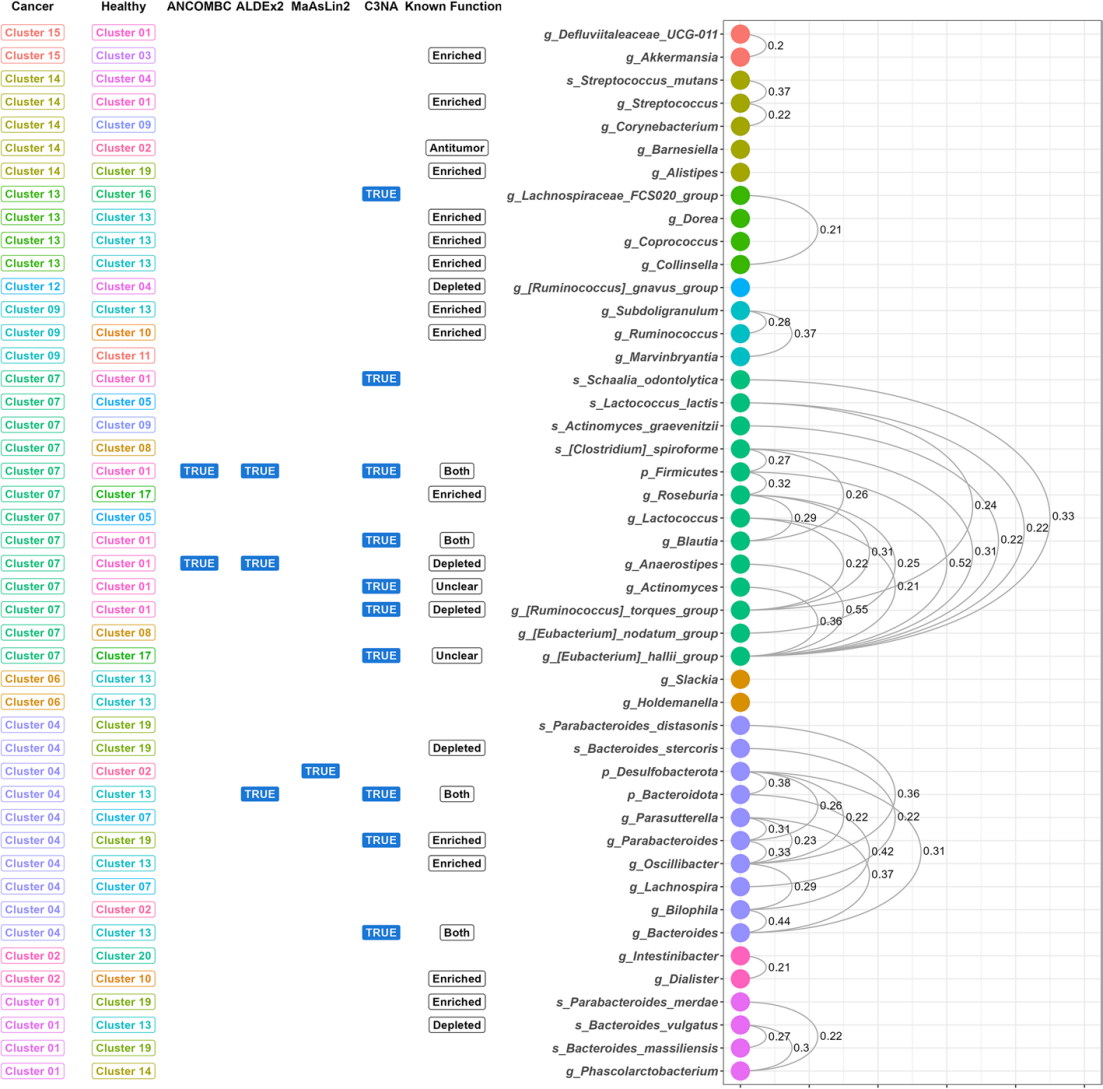
Functional inferences among taxa with disease-only intra-modular correlations. The Colorectal “Cancer” and “Healthy” columns represent the clusters to which the taxa belong if present. The ANCOMBC, ALDex2, and MaAsLin2 with TRUE represent the taxa that are differentially abundant. The C3NA column shows if the taxa are influential in the network between colorectal cancer and healthy control. The known function is obtained from a selection of publications. The disease-only intra-modular correlations identify the important taxa and their corresponding correlation values on the connecting arcs

To further illustrate the usefulness of C3NA to discover unique and solving discrepancies from multiple studies, we extracted known functions from a number of studies and evaluated if the C3NA effectively categorizes the taxa into the correct functional categories. In Fig. 6, we extracted the modular information, DA results, and C3NA disease-only intra-modular correlations from the Baxter et al. analysis and matched them to some of the known functions from the phylum, genus, and species level assignments. Firstly, the DA analyses were unhelpful for this dataset as there are only three genus-level taxa that were labeled as differentially abundance between colorectal cancer and control samples among 27 phylum, genus, or species-level taxa with reported functional roles related to colorectal cancer. Secondly, C3NA was able to group taxa with known and similar functions together and categorize them into the same modules. There is also a clear indication of different healthy (control) categorizations of the taxa groups as indicated by the “Healthy” column in Fig. 6. Lastly, we have expanded the correlation to include additional taxa with disease-only intra-modular correlations with one of the 27 reported taxa, and this approach can be understood as discovery study where potential functions can be assigned to less-studied taxa [14, 42, 43]. For example, Genus *Anaerostipes* [44, 45], which is a probiotic bacteria, is known to deplete among colorectal cancer or adenoma patients, which has a 0.32 correlation with *Aldercreutzia*, which has been recently identified as a microbial biomarker that has more abundance in the Adenoma patients but not so much among colorectal patients by Olovo et al. [46]. Olovo et al. commented on the importance of further studying Aldercreutzia, which produces equol that is associated with a lower risk of colorectal cancer. The similar abundance patterns of these two Genus-level assignments also suggested a similar behavior of them in the colorectal patients, suggesting the potential of an important biomarker to monitor colorectal patients and their disease progression.

Lastly, C3NA enables an interactive comparison of taxa across multiple taxonomic levels between conditions. For instance, Order Burkholderiales, the higher taxonomic level for the genera *Parasutterella* [38] and *Sutterella* [43], both known to be enriched in CRC samples, had an exceptionally high intra-modular correlation with Bacteroides solely in Cancer samples (Fig. 6 and Additional file 1: Fig. S11).

## Discussion

In this paper, we presented a correlation and consensus-based investigation of microbial sequencing data to extract and refine the taxa–taxa co-occurrence network for inferring biological relationships between the microbes. C3NA has a wide range of applications, including detecting specific co-occurrence patterns and identifying, confirming, and assigning functionality to microorganisms. By comparing the co-occurrence patterns that differ between the two conditions, C3NA was able to detect unique microbial patterns that represent condition-specific and study-specific key taxa–taxa interactions. These interactions can be examined further regarding their functional potentials; C3NA can assist in resolving disagreements regarding the contribution of a single microorganism to a given condition by examining the relationship of all its biologically inferable taxonomic categories.

The main advantage of the co-occurrence network approach with the ability to integrate a range of differential abundance analyses is to broaden our understanding of the microbial contribution towards a particular condition. Given the variability of the results from DA methods, C3NA enables the incorporation of as many DA as possible for concordant analyses to extract the most valuable groups of taxa that are differentially abundant or connected between conditions. The functional inference can then be made for the taxa that share high correlations with a well-studied taxon, thus paving the road for discovery studies to validate the potential functions of the less-studied taxa.

In the C3NA pipeline, we utilized on the SparCC algorithms for handling compositional microbial data to output a correlation matrix. SparCC is a validated method for compositional data (e.g., microbiome), and the data do not require any preprocessing, and the resulting taxa–taxa correlations are based on bootstrapped methods with empirical *p*-values for the statistical significance of the inferred taxa–taxa pairs. When incorporating other correlation-based methods, data transformation and normalization techniques suitable for microbial data should also be examined to allow the input data to better represent the composition of the original microbial samples.

In conclusion, we presented a novel microbial data analysis pipeline for enhanced and methodological investigation of microbial communities and their compositional difference between conditions.

## Supplementary Information

**Additional file 1.** This file contains more detailed analyses of the C3NA pipeline, including the SparCC stability and benchmark results, differential abundance results, the impact of taxa filtering between studies, consensus pattern analyses, different correlation-based methods comparison, and the clustering, consensus, and correlation plots from each of the dataset and diseases examined in this paper.

## Data Availability

The two colorectal cancer datasets are PRJNA290926 and PRJEB6070, and the two inflammatory bowel disease datasets are PRJEB13679 and IBDMDB. All raw sequences were downloaded and processed using the same settings. C3NA is available as an R package at https://github.com/zhouLabNCSU/C3NA. Scripts for figures generation are available at https://github.com/zhouLabNCSU/C3NA_ScriptsAndData.

## Abbreviations

OTUs: Operational taxonomic units
ASVs: Amplicon sequencing variants
DA: Differential abundance
SparCC: Sparse correlation for compositional data
TOM: Topological overlap matrix
CRC: Colorectal cancer
CD: Crohn’s disease
