# Supplementary material for: C3NA: correlation and consensus-based cross-taxonomy network analysis for compositional microbial data
Source: BMC Bioinformatics. 2022 Nov 8;23:468. doi: 10.1186/s12859-022-05027-9 (PMC9644555; doi:10.1186/s12859-022-05027-9)
Supplement: Supplementary file 1 — Additional file 1. This file contains more detailed analyses of the C3NA pipeline, including the SparCC stability and benchmark results, differential abundance results, the impact of taxa filtering between studies, consensus pattern analyses, different correlation-based methods comparison, and the clustering, consensus, and correlation plots from each of the dataset and diseases examined in this paper. [file 12859_2022_5027_MOESM1_ESM.docx]

# C3NA – correlation and consensus-based cross-taxonomy network analysis for compositional microbial data

# Additional File 1: Supplementary Results, Tables, and Figures

Kuncheng Song, and Yi-Hui Zhou*

Bioinformatics Research Center, Biological Sciences, North Carolina State University, NC, USA

*To whom correspondence should be addressed

Contact : [yihui_zhou@ncsu.edu](mailto:yihui_zhou@ncsu.edu)

## Supplementary Results

### SparCC Results Comparison between Single-Taxonomic Level and Cross-Taxonomic Levels

C3NA filtered the SparCC results with the taxa-taxa correlations (BH-adjusted p-value ≤ 0.05) and has positive correlations of at least 0.2, which is determined by examining the SparCC’s stability to find correlations from single-taxonomic level SparCC runs.

We benchmarked the comparisons using Baxter et al. with the condition “Cancer”, and each of the runs will include 1,000 iterations of SparCC, and the results are extracted and compared. Firstly, we examine the correlation pairs detected by both single- and multi-taxonomic level runs; the differences are removed and shown in Fig. S1A. The correlation differences between them are primarily between ±0.1. Next, we examined the unique correlations found from either method, as shown in Fig. S1B, and these individual correlations are primarily below 0.2. As a result, we recommended a 0.2 correlation threshold for filtering the taxa-taxa correlation as this is a suitable threshold to detect significant correlations and not too high to omit essential correlations.

### Differential Abundance among Studies

While there are shared taxa identified by different DA methods, there are still disagreements among them, with ANCOM-BC capturing more than the other two methods. C3NA, by definition, captures different taxa as the interpretation of C3NA influential taxa highlights a change in modular membership between the two conditions, not necessarily representing the differentially abundant taxa. Supplementary Fig. S2-5 represents the differential abundance results from Baxter et al., Zeller et al., Gevers et al., and IBDMBD datasets, respectively.

For the ANCOM-BC, we used the Benjamini-Hochberg (BH) adjusted p-value instead of the default Benferroni-Holm methods. The parameter will include the detection of structural zero for better suiting the unique structure of the microbiome data. The determination of the differential abundance taxa is by a BH-adjusted p-value less or equal to 0.05.

For the ALDEx2, we went with the safest approach to maximize the amount of taxa identification from the Wilcoxon output with less than 0.05. Ideally, the best taxa should overlap (95% CI of the effect size omits the null point of zero) and an effect size cutoff of 1. However, both are rare with microbiome data, and the significantly abundant taxa will be defined with a BH-adjusted p-value less or equal to 0.05 from the ALDEx2 output.

For the MaAsLin2, we ran with Arcsine Square Root (AST) transformation without including any covariates. The differential abundance taxa were determined using the q-value, which is calculated using BH adjusted p-value less or equal to 0.05.

### Impact of Filtering on Taxa Identification across Studies and OTUs/ASVs Assignment Methods

In our investigation, we used four different datasets, each undergoing taxonomic assignments via DADA2, two very different methodologies. There are taxa rare taxa across all six taxonomic levels identified and filtered out across studies and Crohn’s disease and Colorectal Cancer. When we compared the remaining taxa across all six levels, we found consistent patterns in which the majority (215) taxa were identified; in addition, the results also highlighted disease-specific and study-specific taxonomic assignments (Supplementary Fig. S6). We also investigated the removed taxa, and their patterns are unique to the study and the clustering methods (Supplementary Fig. Sd7). The number of filtered taxa and original taxa are shown in Supplementary Table 1, and this reduction of rare taxa corresponds to a decrease in computation time, which used 12 cores on Intel(R) Xeon(R) CPU E5-2670 0 @ 2.60GHz with 30 GB of RAM.

### Effect of Consensus-based on Different pattern selections

When we examined the module membership changes from different minimal module sizes, there is a clear trend of decreasing changes in module memberships, and as we reduce the number of modules below ten, there will be more duplicated patterns (Fig. S1-S12). We selected all unique designs with minimal module sizes equal to or greater than ten for all our investigations, providing consistent results.

We examine the impact of different module patterns and the optimal number of clusters based on the consensus matrix by examining the result of remaining significant correlations after clustering. Using different selected module patterns and a range of optimal clusters, we evaluated how many key correlations remain. The results show consistency in important correlations, particularly for the essential correlations greater or equal to 0.2. We determine the final taxa cluster through clustering of the consensus matrix (Fig. S12-S19), there is a clear trend of a quick increase of average silhouette widths with the increased number of clusters, and it gradually plateaued after 15 clusters. As Supplementary Fig. S9 show, we can generally categorize the silhouette plot into three categories, the first panel is the “base dynamic region” (minimal number of taxa per module from 3 to 12), where we include all the dynamically changing regions, and with more patterns, the silhouette width plot gradually forms a curve with a plateau region from linear trends. The second panel includes the “less dynamic region,” which includes the region with a few duplicated patterns, but the pattern generally changes (small changes) with each increment (the minimal number of taxa per module ranges from 13 to 25). The third panel includes the “stable region” where the majority of repetitive patterns occur. C3NA recommends the user to pick any of the dynamic regions which can be identified by minimal repetitive region with the number of modules for each pattern between 10 to 20. As the first and third panel indicates selections, other regions might lead to an unstable silhouette plot for the optimal number of cluster determination.

Next, we evaluated the impact of the remaining number of significant intra-modular correlations greater or equal to 0.2 from each of the parameters we observed with a similar number of clusters with an optimal number of clusters greater than 15. This also indicates the stability of the consensus-based clustering; with enough patterns selected, slight differences in a few patterns and different numbers of optimal clusters will not drastically affect the downstream correlation results. For example, when we look at the minimal number of taxa per module from 15 to 22 (Supplementary Fig. S9B, x-axis), after the proportion of zeros per cluster dropped below 10% (uncrossed-off numbers), the number of intra-modular correlations between the optimal number of clusters 15 – 22 are all around 1,300 ± 100. C3NA advises using an optimal number of clustering as low as possible once the silhouette plot enters the plateau region.

### SparCC Benchmark

SparCC algorithms enable parallel programming, but they can be computationally expensive in both storage requirements and time. The computation time varies depending on the number of taxa extracted as well the number of strong correlations presented at each bootstrap, and 100 bootstraps can take a few hours to run under multi-core settings. Time consumption for each of the examined condtions is recorded in Supplementary Table 1. Also, we investigated the impact of using fewer iterations, and the results indicated smaller number of iterations would have more significant correlations, most of which are below 0.3 (Supplementary Result). For preliminary investigation, it is possible to run as little as ten iterations, and the user is advised to adjust the display on the Shiny application for the correlation to increase the correlation cutoff to 0.3.

### SparCC Stability with Different Number of Bootstraps

We use the DADA2 ASV assignment method with the Cancer samples from Baxter et al. to evaluate the impact of iterations on the stability of SparCC (Supplementary Fig. S11)^1^. For each of the 10, 25, 50, 100, and 500 iterations, we run six different bootstrap rounds. We investigate the difference in terms of taxa-taxa correlations greater or equal to 0.2 with BH-adjusted p-values less or equal to 0.05. The results show that most of the correlations are shared compared to 1,000 iterations. The most significant difference is the total number of these correlation pairs, with 1,000 iterations having roughly 16% fewer correlations compared to that with ten iterations, though most of the missed correlations are less than 0.3. Thus, when running a smaller number of iterations for preliminary investigation and evaluation under the Shiny application, the user should filter the correlations to 0.3. We also evaluated the time consumption while running these bootstraps. The computation time for 10, 25, 50, 100, and 500 using one core on Intel(R) Xeon(R) CPU E5-2670 0 @ 2.60GHz are 10 minutes, 1.25 hours, 3 hours, 10 hours, and 34 hours, respectively.

### Comparison of Different Correlation Methods

For our C3NA, we focus mainly on the SparCC algorithm as it is one of the first and most used correlation-method for handling compositional data. Here, we compare the differences in terms of pairwise correlation differences among SparCC, COAT^2^, and Pearson^3^. For both COAT and Pearson, we convert the raw stacked matrix into the compositional format by normalizing the taxa by dividing each taxon by the sum of corresponding taxonomic levels per sample. These data will be used as the input for COAT and Pearson data. Moreover, we filtered to positive correlation values in one of the methods since negative correlations are not treated as no correlation under the signed TOM Similarity settings. Among these positive correlation pairs, the Pearson method generates considerably different results from both COAT and SparCC (Supplementary Fig. S12A). This is expected as Pearson is not designed for compositional data.

Next, we compare the COAT and SparCC results, which are highly correlated (r = 0.904). Because of the difference in the algorithms, the distribution of taxa-taxa correlations will be slightly different. Fig. S12A scatter plot between SparCC and COAT illustrates the similarity and differences between the taxa-taxa correlations, and when the correlations are greater than 0.25, with a given SparCC (x-axis), the corresponding COAT (y-axis) correlations tend to be greater. Hence, it is recommended for the user to choose a smaller correlation cutoff lower than 0.2 (the suggested minimal cutoff for the SparCC method) and utilize the Shiny application to filter the correlations before increasing this minimal cutoff value gradually. In addition, because of this difference in the correlation distributions, we will use a percentile cut to compare the top 900 taxa-taxa pairs, which include both intra-modular and inter-modular pairs (Supplementary Table 2). These should represent the high correlation taxa-taxa pairs and should be the key correlations for investigating the similarity of top taxa-taxa pairs from different correlation methods in the presence or absence of DESEq2 normalization. The majority of these taxa-taxa pairs are shared between the SparCC and COAT regardless of the normalization, which also covers most of the highly correlated taxa-taxa pairs. There are discrepancies among the results due to the different distributions and module selections between these combinations among both Cancer and Control samples (Supplementary Fig. 13 A and B). We further compared the difference between the SparCC and COAT in the presence and absence of DESeq2 normalization, and the results suggest very similar results (Supplementary Fig. 14 A and B).

Overall, additional validation needed to be performed when switching to a different normalization, transformation, and correlation method.

### Optimal Number of Clusters Selection for Datasets included in the study

The patterns and silhouettes curves for the datasets are saved on GitHub (<https://github.com/zhouLabNCSU/C3NA_ScriptsAndData>) under the file Supplementary Figures.docx with Fig. S12 – S19.

### Consensus Matrices for the Datasets included in the study

The consensus matrices based on the optimal number of clusters are saved on GitHub (<https://github.com/zhouLabNCSU/C3NA_ScriptsAndData>) under the file Supplementary Figures.docx with Fig. S20 – S27.

### Correlation Matrices for Datasets included in the study

The correlation matrices based on the optimal number of clusters are saved on GitHub (<https://github.com/zhouLabNCSU/C3NA_ScriptsAndData>) under the file Supplementary Figures.docx with Fig. S28 – S35.

**Reference**

1. Callahan, B. J. *et al.* DADA2: High-resolution sample inference from Illumina amplicon data. *Nat. Methods* **13**, 581–3 (2016).

2. Cao, Y., Lin, W. & Li, H. Large Covariance Estimation for Compositional Data Via Composition-Adjusted Thresholding. *https://doi.org/10.1080/01621459.2018.1442340* **114**, 759–772 (2018).

3. K., P. On a form of spurious correlation which may arise when indices are used in the measurement of organs. *R. Soc., London, Proc.* **60**, 489–502 (1897).

4. Love, M. I., Huber, W. & Anders, S. Moderated estimation of fold change and dispersion for RNA-seq data with DESeq2. *Genome Biol.* **15**, 550 (2014).


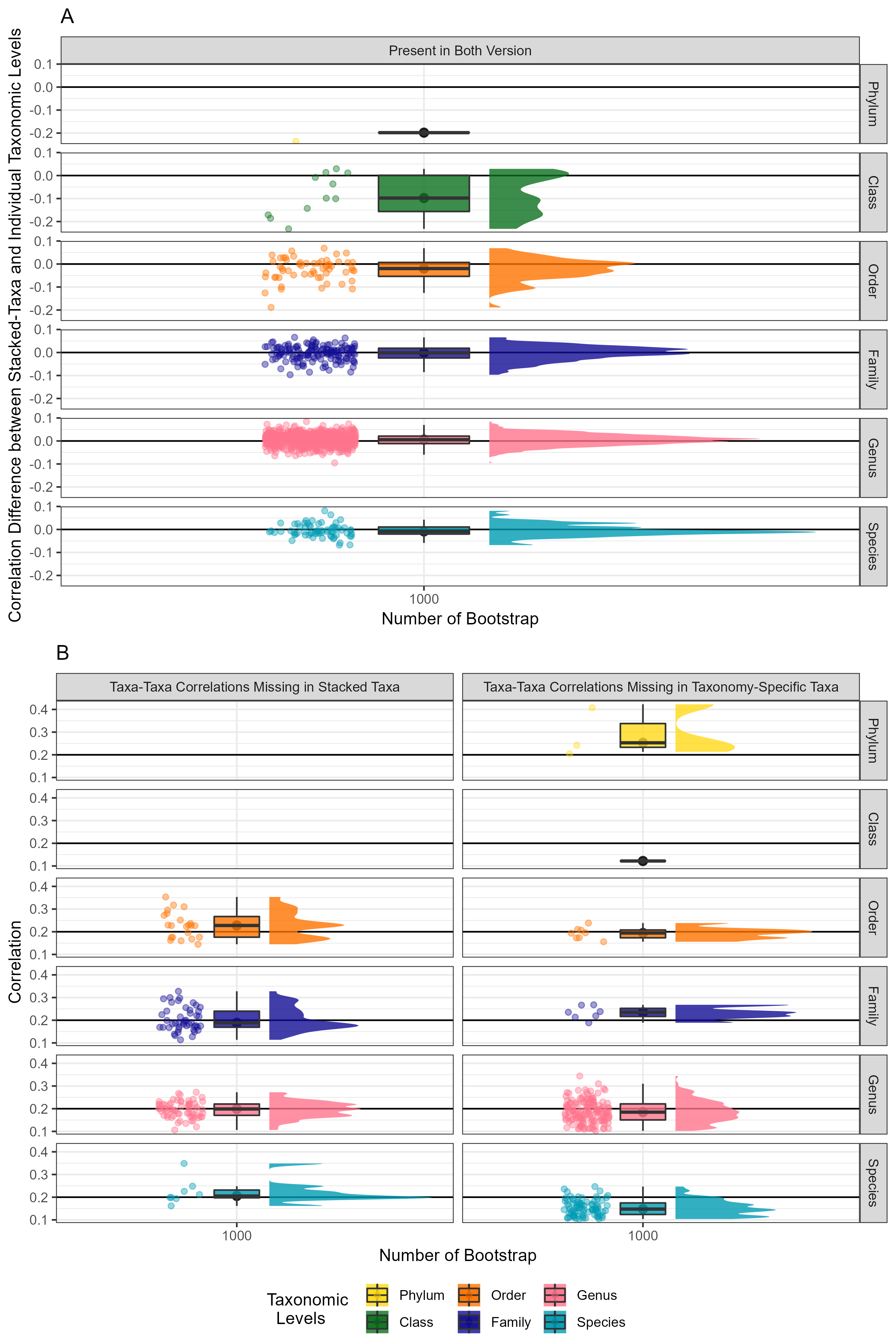


**Supplement Figure 1.** Comparison of Stacked-Taxa Correlation with Individual Taxonomic Correlation. The results used the Colorectal Cancer from Baxter et al., with the samples associated with the condition “Cancer.” (**A**) Comparison of the difference between the stacked-taxa with the individual taxonomic correlations for the taxa-taxa pairs that are above 0.1 with adjusted p-values less or equal to 0.05. (**B**) Comparison of the stacked-taxa or individual taxonomic only correlations that do not present in the other results.


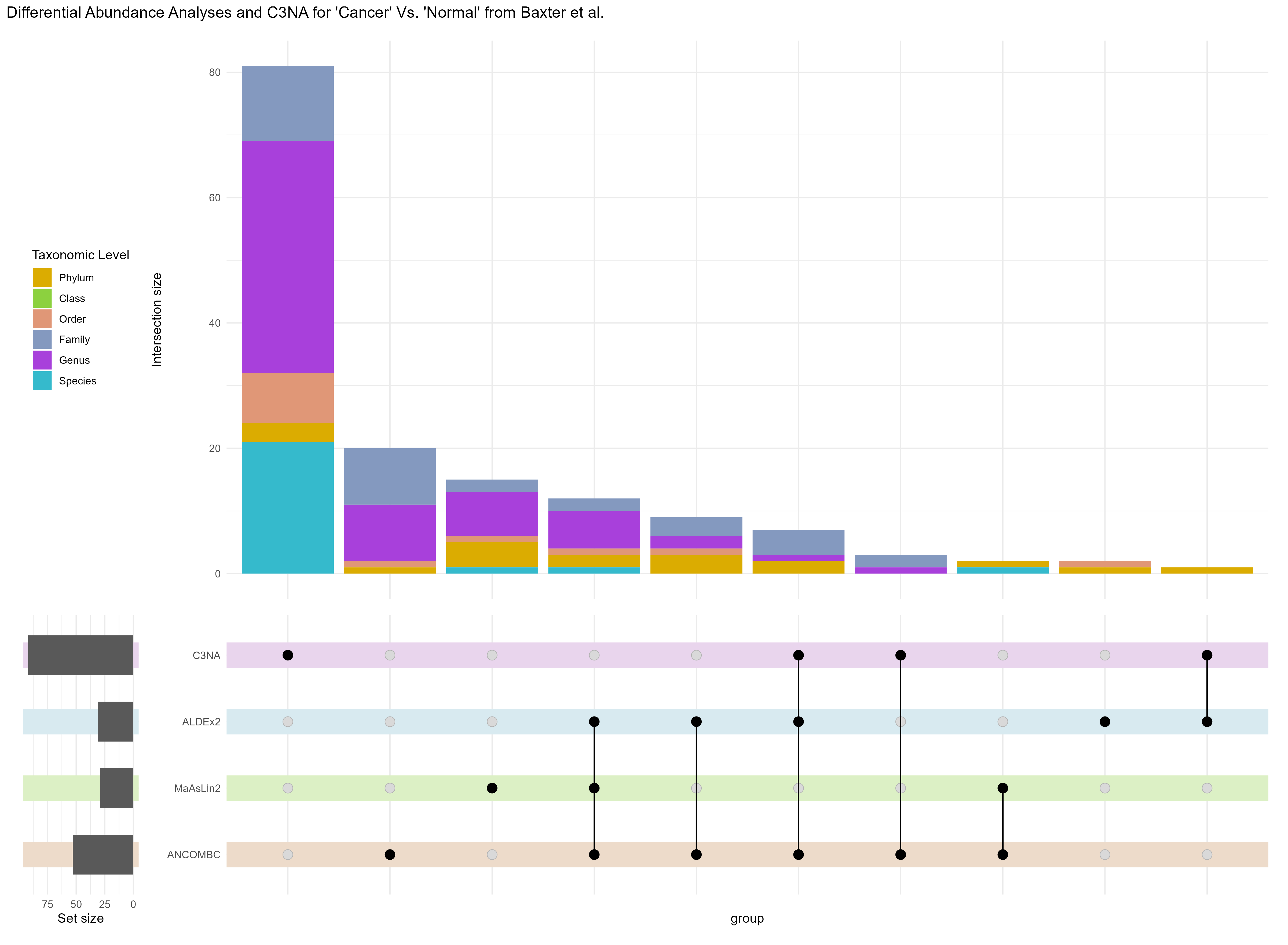


**Supplement Figure 2.** Compare differentially abundant taxa between the “Cancer” and “Normal” among two OTUs/ASVs assignment methods in Baxter et al. Orange represent ANCOM-BC, green represents MaAsLin2, blue represents ALDEx2, and purple represents C3NA influential taxa.


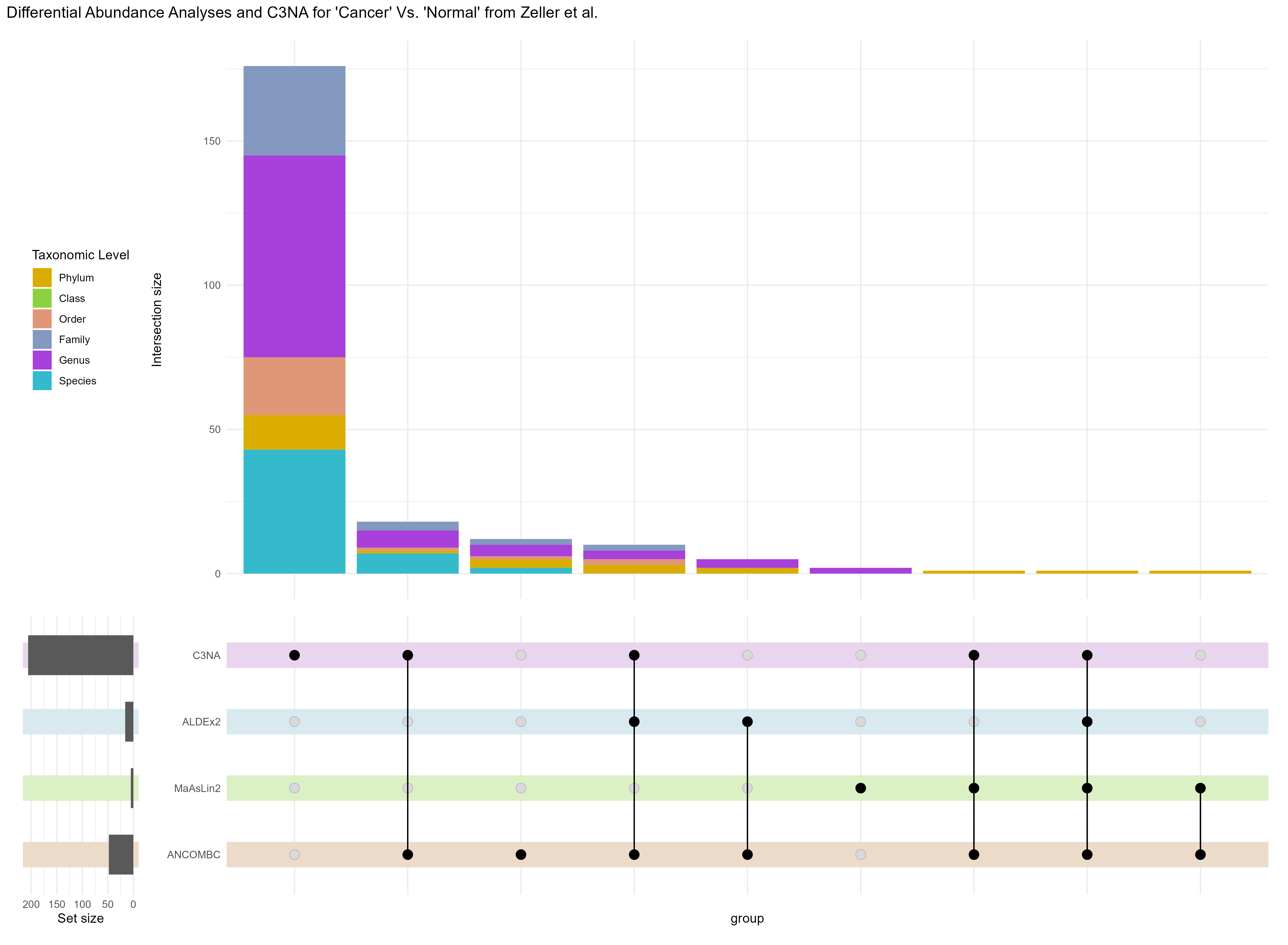


**Supplement Figure 3.** Compare differentially abundant taxa between the “Cancer” and “Normal” among two OTUs/ASVs assignment methods in Zeller et al. Orange represent ANCOM-BC, green represents MaAsLin2, blue represents ALDEx2, and purple represents C3NA influential taxa.


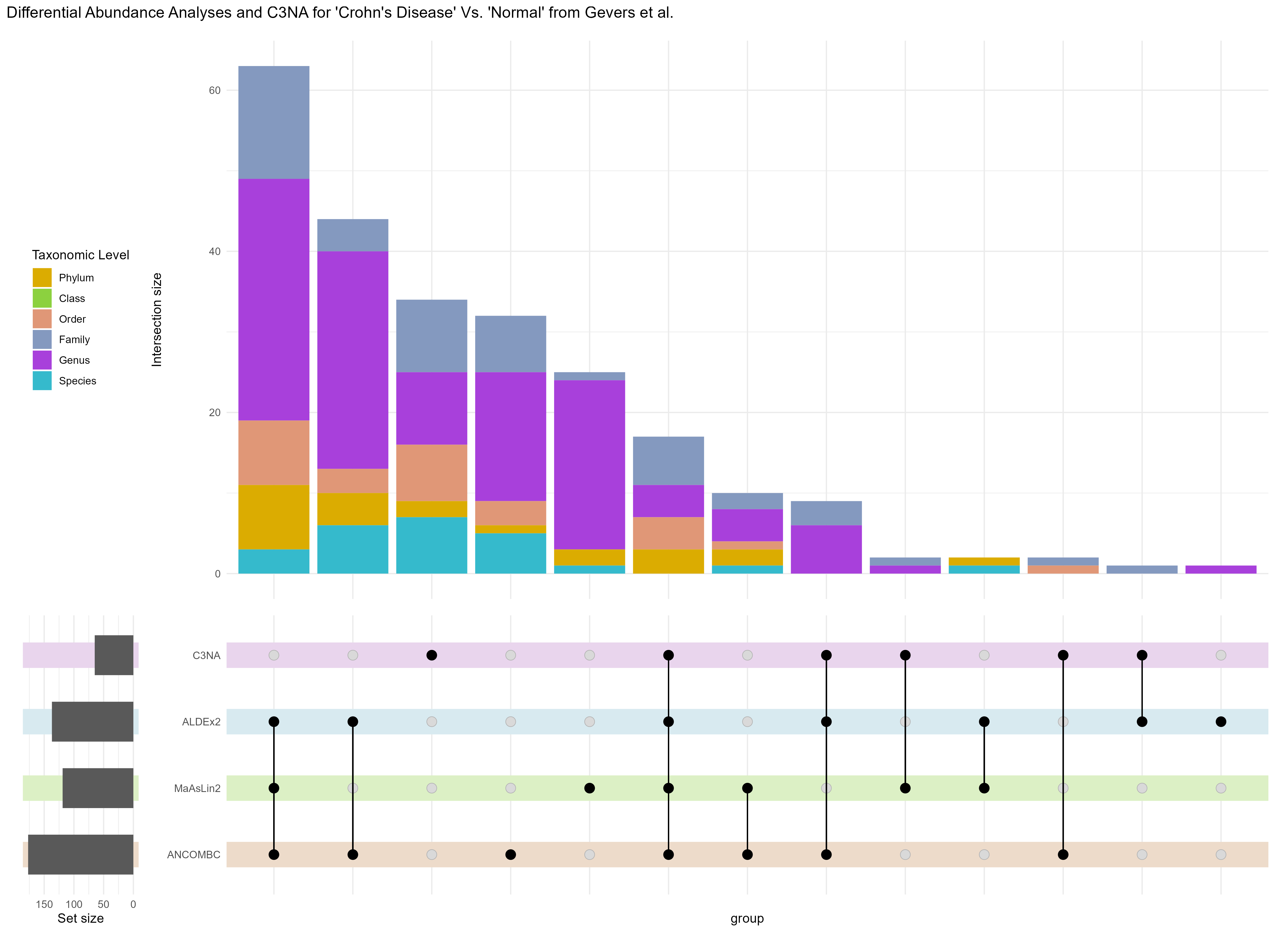


**Supplement Figure 4.** Compare differentially abundant taxa between the “Cancer” and “Normal” among two OTUs/ASVs assignment methods in Gevers et al. Orange represent ANCOM-BC, green represents MaAsLin2, blue represents ALDEx2, and purple represents C3NA influential taxa.


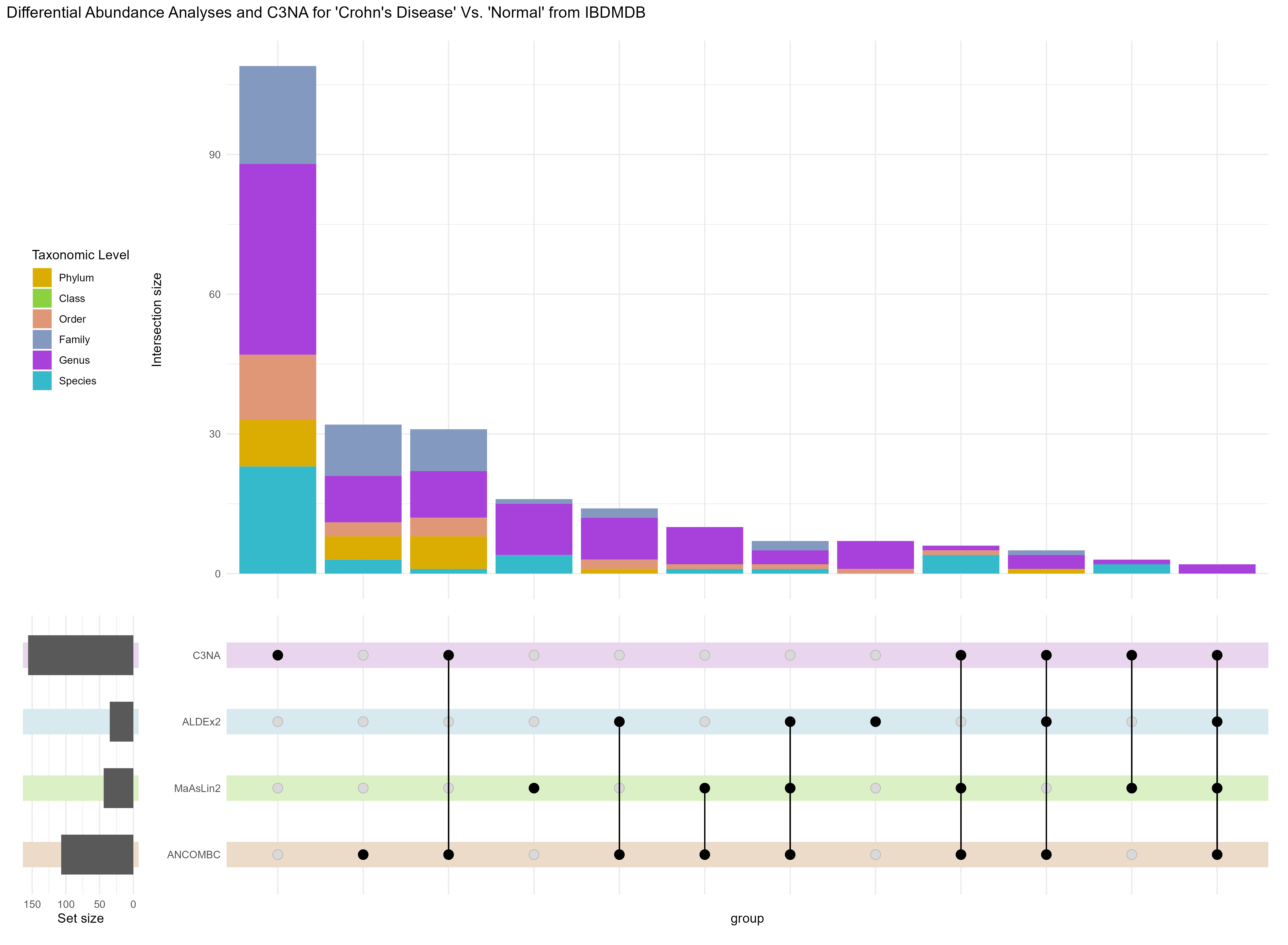


**Supplement Figure 5.** Compare differentially abundant taxa between the “Cancer” and “Normal” among two OTUs/ASVs assignment methods in IBDMDB. Orange represent ANCOM-BC, green represents MaAsLin2, blue represents ALDEx2, and purple represents C3NA influential taxa.


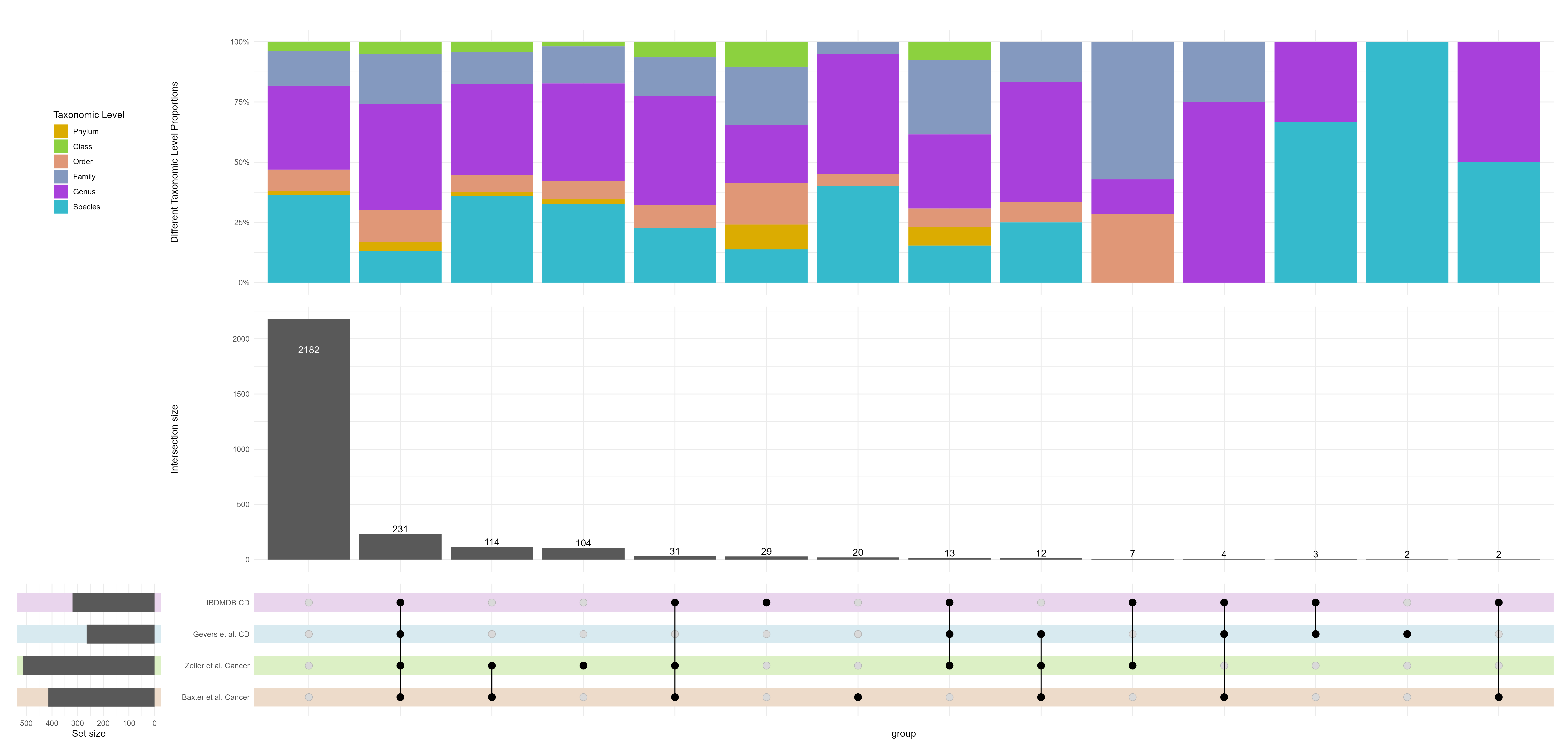


**Supplement Figure 6.** Impact of Filtering on Taxa Identification across Studies and OTUs/ASVs Assignment Methods from Disease. The top barplot highlights the proportion of each taxonomic level within the corresponding intersected taxa. The bottom bar plot highlights the intersected taxa with the solid point representing the presence of taxa in the corresponding row combination of study, clustering methods, and condition. Conditions: CD: Crohn’s disease; Cancer: Colorectal Cancer.


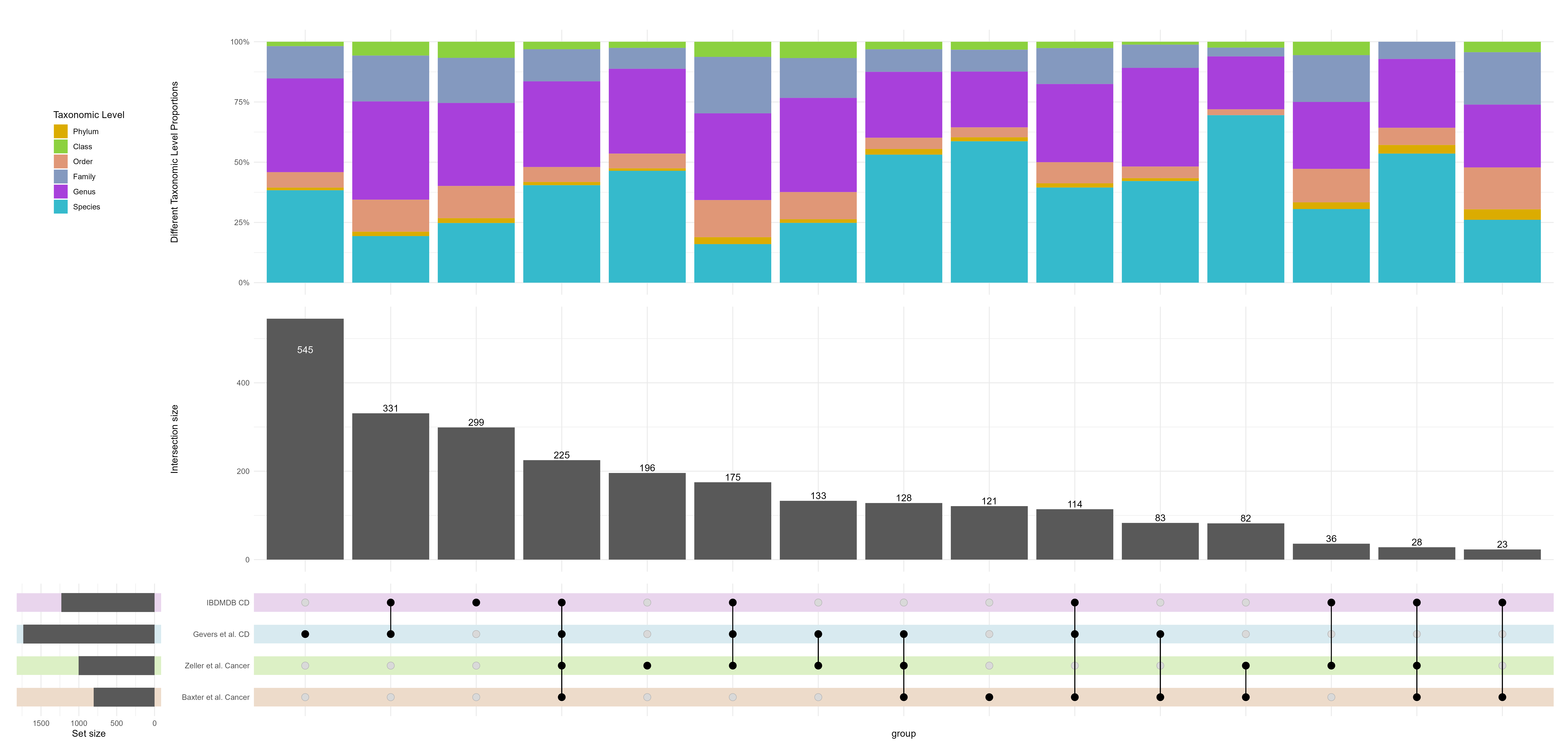


**Supplement Figure 7.** Removed Taxa from Six Levels across Studies and OTUs/ASVs Assignment Methods from Disease. The top barplot highlights the proportion of each taxonomic level within the corresponding intersected taxa. The bottom bar plot highlights the intersected taxa with the solid point representing the presence of taxa in the corresponding row combination of study, clustering methods, and condition. Conditions: CD: Crohn’s disease; Cancer: Colorectal Cancer.





**Supplement Figure 8. A.** Average silhouette width plots with all Minimal Number of Taxa per Module and with Different Number of Clusters. **B.** Effects of Intra-modular Correlations from Different Minimal Number of Taxa per Module and Optimal Number of Clusters. The cross-out numbers represent the number of intra-modular correlations collected from clusters, including more than 10% intra-modular consensus proportions. The data is generated using the Baxter et al. with “Cancer” samples under the DADA2 ASV assignment method, and the minimal number of taxa per module chosen with ten modules is 21, and the optimal number of clusters chosen is 15. The corresponding consensus and correlation plots are Fig. S2 and S13, respectively. The white circle includes the ideal region for selection.


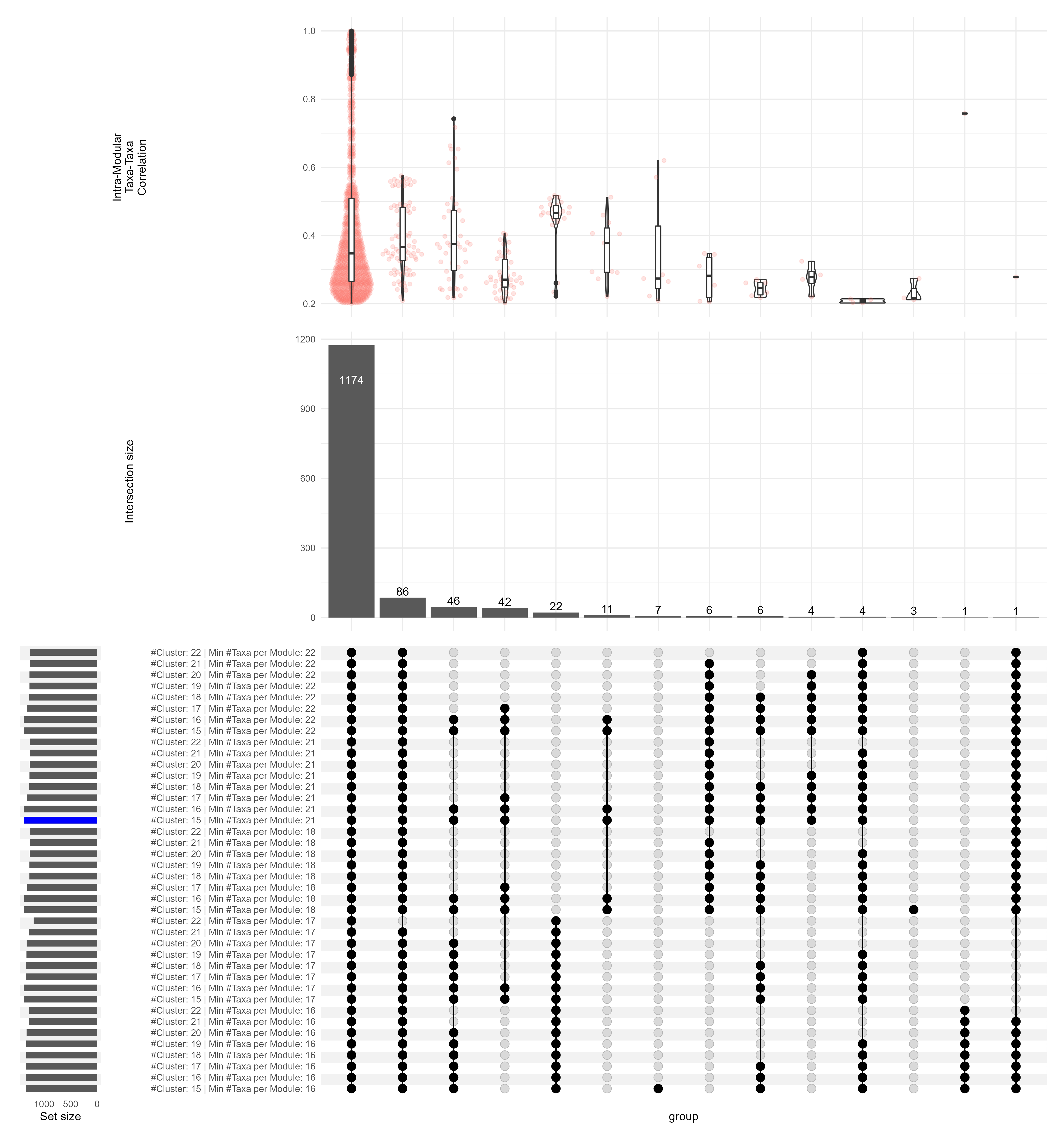


**Supplementary Results Figure 9.** Intra-modular taxa-taxa correlations are shared among the selected combination of Minimal Number Taxa per Module and Number of Clusters. The blue bar highlighted set represents the selected combination for C3NA analysis.


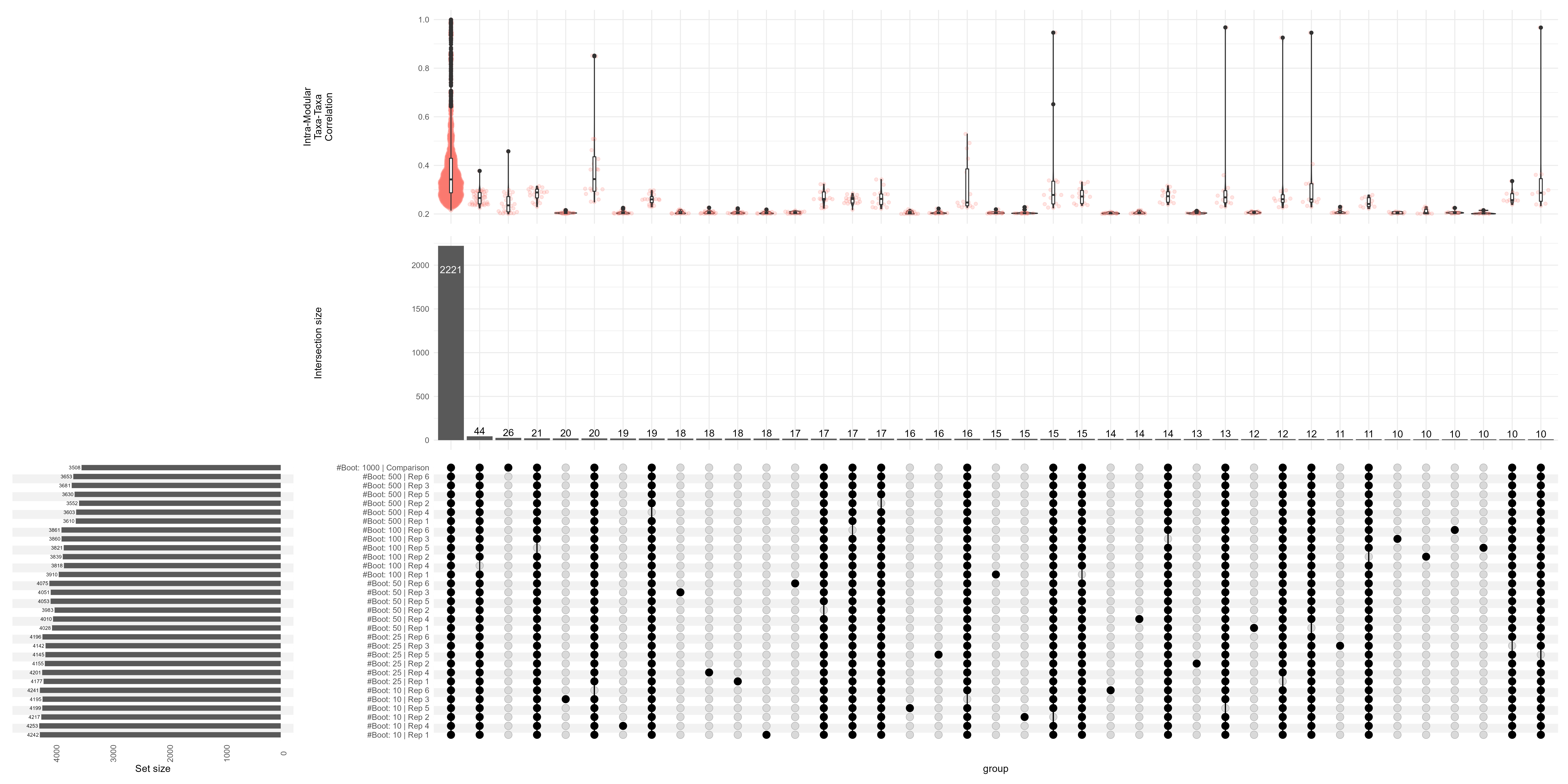


**Supplementary Results Figure 10.** Comparison of Significant Taxa-Taxa Correlations from Different Number of Bootstraps in sparCC. Each bootstrap undergoes six different replications. The number of bootstraps includes 10, 25, 50, 100, 500, and these are compared to the standard 1,000 iterations. Intersect taxa with less than ten taxa are removed, and the boxplot on the top represents the average correlation with each intersected taxa-taxa correlation among the different combinations.


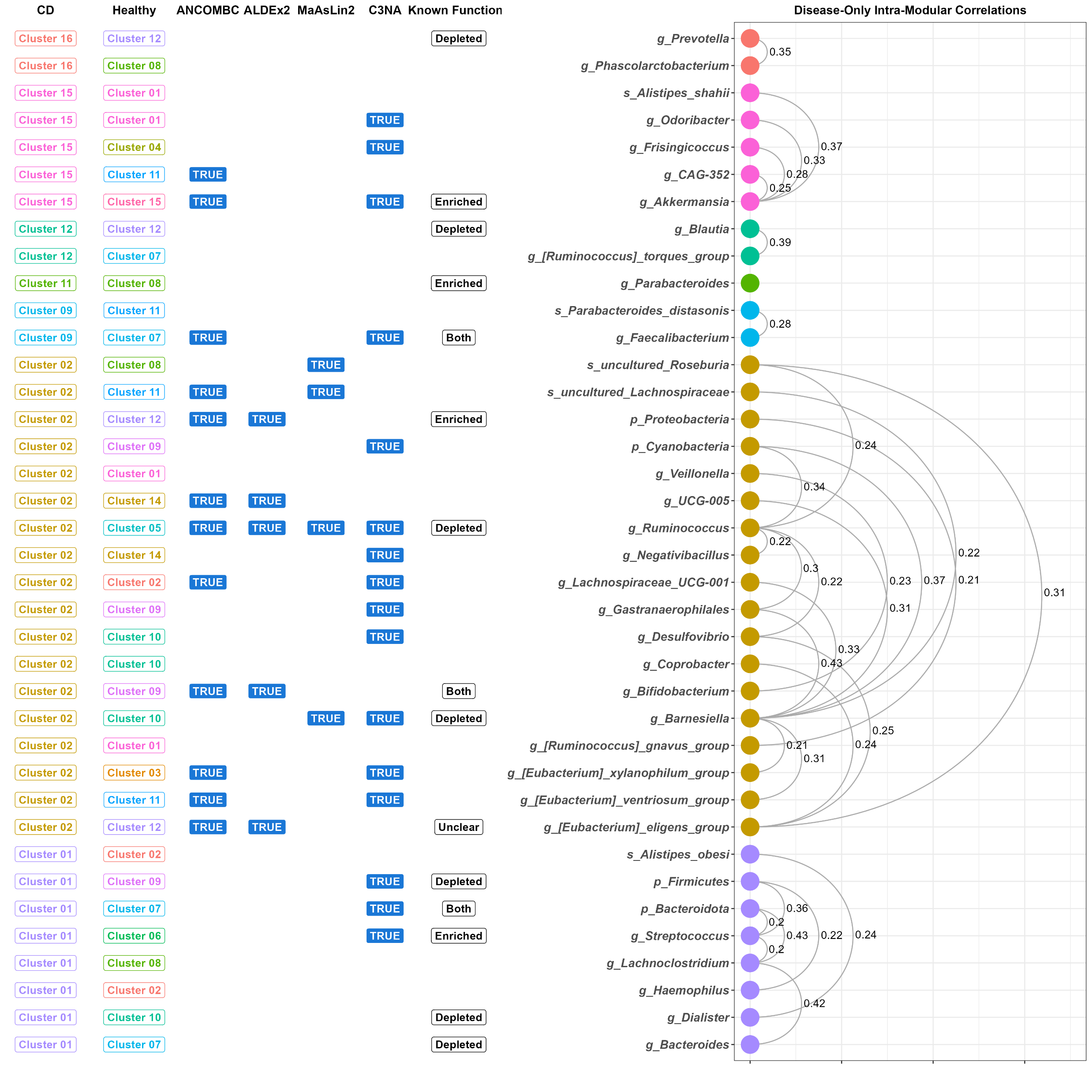
**Supplementary Results Figure 11.** Functional inferences among taxa with disease-only intra-modular correlations. The Crohn’s and Healthy columns represent the cluster to which the taxa belongs if present. The ANCOMBC, ALDex2, and MaAsLin2 with TRUE represent they are differentially abundant. The C3NA column shows if the taxa are influential in the network between disease and control. The known function is obtained from a selection of publications. The disease-only intra-modular correlations identify the important taxa and their corresponding correlation values on the connecting arcs. Genera


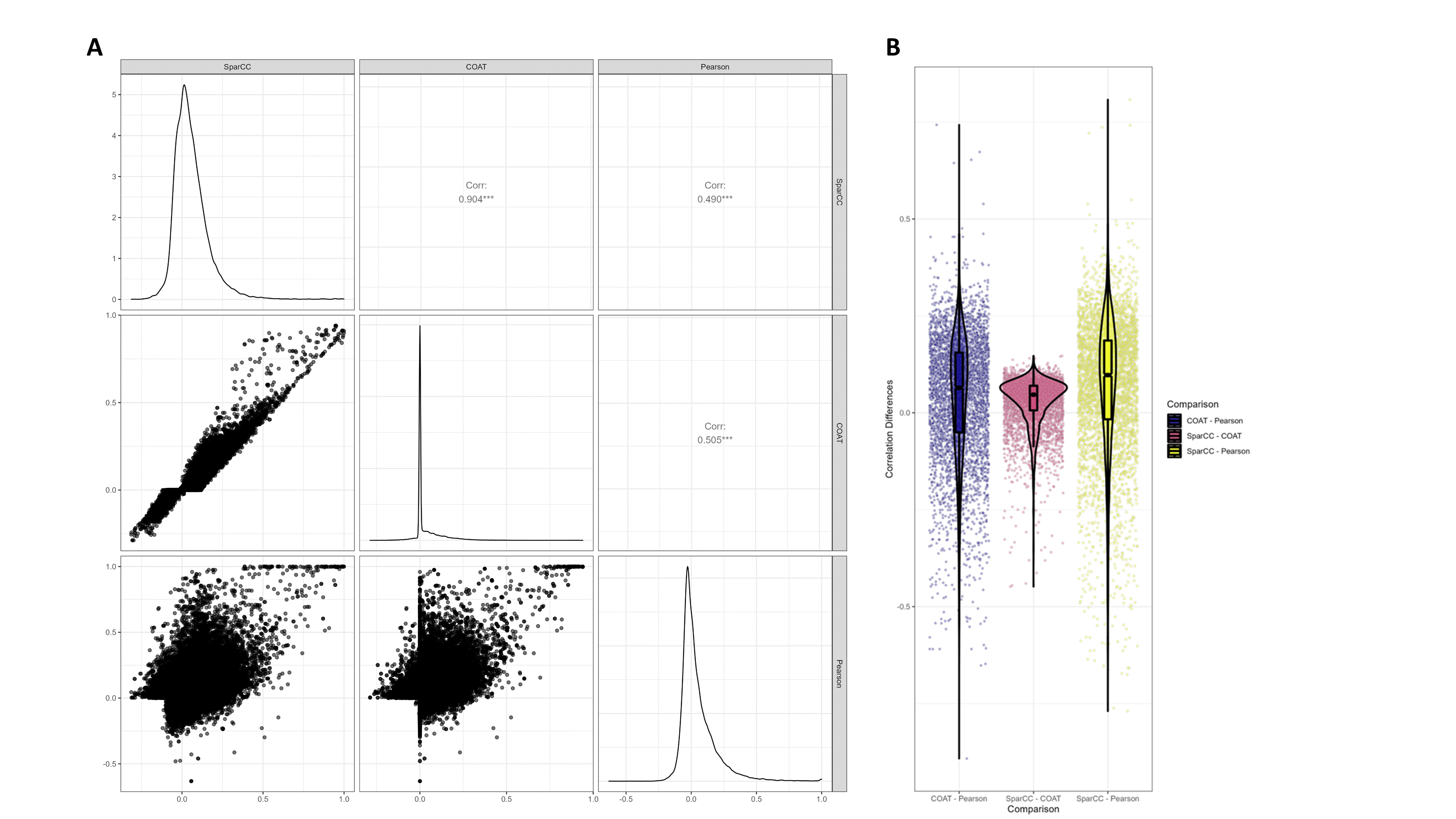


**Supplementary Results Figure 12.** Functional inferences among taxa with disease-only intra-modular correlations. **A.** Comparison between the three correlation methods using Ggallery R Package. Lower triangle plots represent the pairwise correlation scatter plot between the methods, and these pairs only include the taxa-taxa pairs that have at least a positive correlation in one of these three methods. Diagonal plots are the density plot for each of the methods. Upper triangle plots are the Pearson Correlation test score, *** indicates a p-value < 0.001. **B.**  Comparison between the correlation difference between the COAT, Pearson, and SparCC. The taxa-taxa have been filtered to correlations with at least positive among one of these three methods.


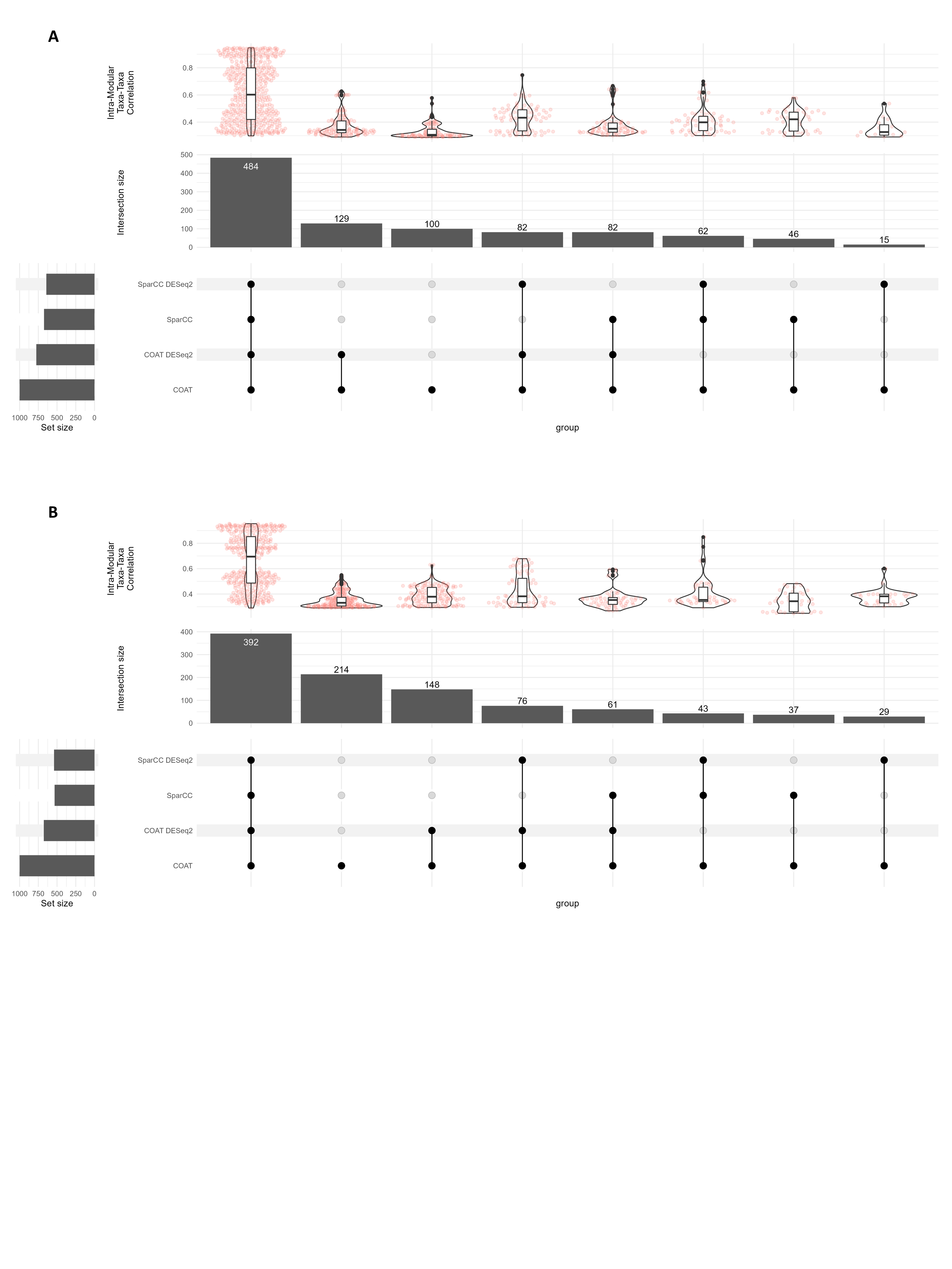
**Supplementary Results Figure 13.** Comparison of Top 1,000 taxa-taxa correlation pairs from SparCC and COAT with and without DESeq2 Normalization. All tests were made using the Baxter et al. data. **A.** Phenotype: Cancer. **B.**  Phenotype: Control.


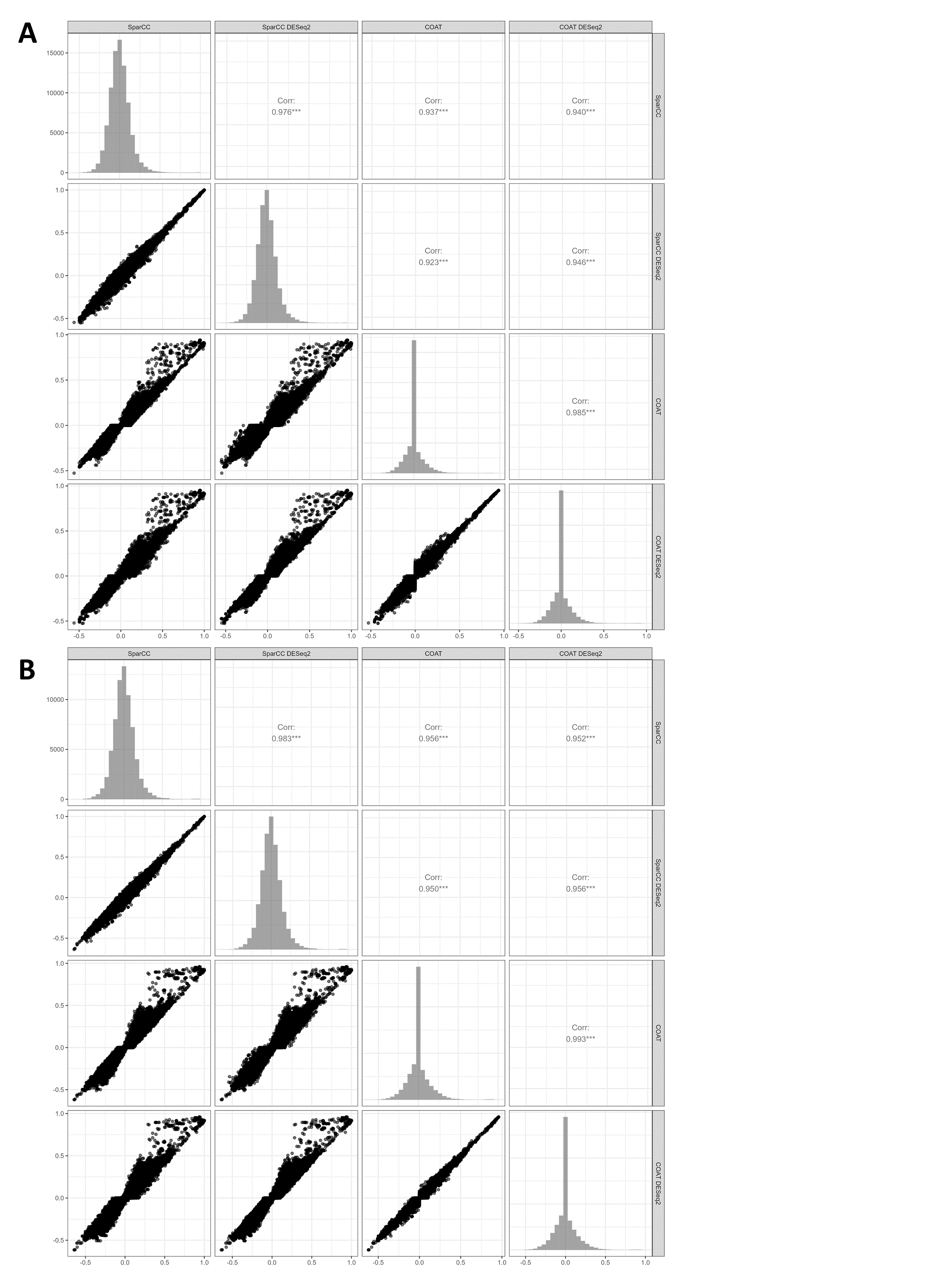


**Supplementary Results Figure 14.** Pairwise comparisons between the COAT and SparCC with and without DESeq2 normalization. Tests were made using the Baxter et al. data. Lower triangle plots represent the pairwise correlation scatter plot between the methods. Diagonal plots are the histogram for each of the methods. Upper triangle plots are the Pearson Correlation test score, *** indicates a p-value < 0.001. **A.** Phenotype: Cancer. **B.**  Phenotype: Control.

|  | | | | | | | | **C3NA** | | | **Differential Abundance** | | |
| --- | --- | --- | --- | --- | --- | --- | --- | --- | --- | --- | --- | --- | --- |
| **Dataset** | **Condition** | **Number of Samples** | **Unfiltered Taxa** | **Number of Filtered Taxa** | **Taxa-Taxa Correlation** | **Taxa Remaining Percentage (%)** | **SparCC Computation Time ( hours )** | **Number of Modules** | **Intra-Modular Taxa-Taxa Correlation** | **Influential Taxa** | **ANCOM-BC** | **ALDEx2** | **MaSaLin2** |
| **Baxter et al.** | Cancer | 127 | 954 | 414 | 3,885 | 43.40% | 122 | 15 | 1,549 | 92 | 53 | 31 | 29 |
|  | Control | 134 | 839 | 372 | 4,314 | 44.34% | 96 | 20 | 1,014 |  |  |  |  |
| **Zeller et al.** | Cancer | 41 | 921 | 512 | 3,219 | 55.59% | 125 | 23 | 2,405 | 206 | 51 | 21 | 5 |
|  | Control | 50 | 932 | 502 | 3,011 | 53.86% | 125 | 15 | 2,580 |  |  |  |  |
| **Gevers et al.** | CD | 731 | 1634 | 265 | 2,801 | 16.22% | 47 | 13 | 1,045 | 65 | 177 | 137 | 119 |
|  | Control | 335 | 1429 | 296 | 2,805 | 20.71% | 104 | 22 | 839 |  |  |  |  |
| **IBDMDB** | CD | 86 | 1148 | 320 | 4,383 | 27.87% | 124 | 16 | 1,376 | 156 | 107 | 35 | 44 |
|  | Control | 46 | 859 | 355 | 2,878 | 41.33% | 115 | 15 | 1,742 |  |  |  |  |

**Supplementary Table 1.** Dataset summaries from the four studies and two conditions

| **Correlation Method** | **Transformation Method** | **Phenotype** | **Number of Taxa** | **Total Number of Unique Taxa-Taxa Pairs** | **Number of Optimal Modules** | **Correlation Cutoffs from Top 1,000 Taxa-Taxa Pairs*** | **Sum of Intra-Modular and Inter-Modular based on the Correlation Cutoffs** |
| --- | --- | --- | --- | --- | --- | --- | --- |
| **SparCC** | None | Cancer | 414 | 85,491 | 15 | 0.3316 | 1,440 |
|  | DESeq2 |  |  |  | 18 | 0.3217 | 1,494 |
| **COAT** | None |  |  |  | 18 | 0.3288 | 2,424 |
|  | DESeq2 |  |  |  | 22 | 0.3216 | 2,198 |
| **SparCC** | None | Control | 372 | 69,006 | 20 | 0.3368 | 1,046 |
|  | DESeq2 |  |  |  | 20 | 0.3418 | 1,332 |
| **COAT** | None |  |  |  | 16 | 0.2604 | 1,984 |
|  | DESeq2 |  |  |  | 15 | 0.3127 | 2,222 |

*The top 750 taxa-taxa pairs only contain the intra-modular pairs

**Supplementary Table 2.** Transformation Evaluations on Top 1,000 Taxa-Taxa Pairs from each Combinations
